# Development of a neutron texture analysis reduction pipeline in *Mantid*

**DOI:** 10.1107/S1600576726007661

**Published:** 2026-09-28

**Authors:** Andy Bridger, Winfried Kockelmann, Joe Kelleher, Manuel Morgano, Richard Waite, Sarah Foxley, Florencia Malamud

**Affiliations:** a ISIS Facility STFC-Rutherford Appleton Laboratory Didcot OX11 0QX UK; b Laboratory for Neutron Scattering and Imaging (LNS) Paul Scherrer Institut (PSI) 5232 Villigen PSI Switzerland; Montanuniversität Leoben, Austria

**Keywords:** neutron texture analysis, engineering diffraction, data reduction, *Mantid*

## Abstract

We present the implementation of an experimental planning and data analysis pipeline for spatially resolved neutron texture analysis within the *Mantid* framework. The workflow processes time-of-flight neutron diffraction data to generate corrected experimental pole figures suitable for quantitative texture analysis of intact engineering components. It provides integrated tools for texture measurement planning, instrument calibration and experimental pole-figure generation. Pole figures and orientation distribution functions (ODFs) are produced in formats compatible with standard texture analysis software such as *MTEX*, enabling streamlined quantitative texture characterization across neutron facilities. The workflow is validated using an AA7150 aluminium dataset previously analysed with *NyRTeX*, showing consistent pole figures and ODF-derived texture components while enabling flexible detector grouping and acquisition-time studies within *Mantid*.

## Abstract 2

An experimental planning and data processing pipeline for spatially resolved neutron texture analysis has been implemented within the *Mantid* framework. The workflow processes time-of-flight neutron diffraction data to generate experimental pole figures, enabling non-destructive quantitative texture analysis of materials and components with complex texture distributions.

## 1. Introduction

Texture is one of the most important material parameters in engineering and earth sciences for describing and understanding the anisotropy of polycrystalline materials. In polycrystals, such as many engineering alloys, ceramics and rocks, individual grains are rarely randomly oriented. Instead, they often exhibit statistical preferred crystallographic orientations. Crystallographic texture of polycrystalline materials is an important source of structural anisotropy that leads to direction-dependent physical and mechanical properties and macroscopic responses that are highly relevant in industrial (Engler, 2025 ▸), technological (Zuern *et al.*, 2026 ▸) and geo­logical contexts (Kocks *et al.*, 2000 ▸). Texture within a polycrystalline material affects a wide range of properties, including formability, strength, elastic stiffness, electrical and thermal conductivity, and magnetic anisotropy. Understanding the distribution of grain orientations is therefore essential for predicting how materials respond to external forces, such as mechanical loading, thermal gradients or magnetic fields (Wenk & Van Houtte, 2004 ▸). In metallurgy, this understanding is essential for the development and control of materials under well defined experimental processing conditions to achieve specific properties. In earth sciences, geological textures of rock-forming minerals serve as fingerprints of a geological history and must be considered for geophysical modelling and prospecting (Schaefer, 2002 ▸).

Texture describes the distribution of crystallographic orientations in a polycrystalline material. Diffraction techniques such as X-ray, electron and neutron diffraction are commonly used to determine the orientation distribution function (ODF) of grains in polycrystalline samples (Bunge, 1982 ▸). Owing to the relatively weak interaction of neutrons with most metals and minerals, neutron diffraction offers penetration depths significantly greater than those of X-rays or electrons. This makes it particularly suitable for non-destructive investigations of large sample volumes and complex engineering components, providing measurements that are representative of the bulk material. Neutron diffraction is advantageous for materials with large grain sizes where other diffraction techniques may suffer from poor statistical sampling. Neutron texture diffraction therefore provides an important complement to laboratory or synchrotron X-ray diffraction and electron backscatter diffraction (EBSD), which offer high spatial resolution and rapid data acquisition but are generally limited to surface or near-surface regions due to their much smaller penetration depths (Wenk & Van Houtte, 2004 ▸). In addition, neutron diffraction enables *in situ* studies during mechanical loading or phase transformations, allowing investigation of microstructural evolution and deformation mechanisms. Because neutron measurements are non-destructive and do not require sampling, texture analysis can also be applied to complex industrial components and irreplaceable archaeological or cultural heritage objects (Kockelmann *et al.*, 2006*b* ▸; Malamud *et al.*, 2017 ▸). In both engineered and geological materials, crystallographic texture provides important information on processing history and deformation mechanisms. For archaeological metals, texture analysis reveals the deformation history, for example whether or not a metal object was mechanically worked, cast or annealed. In geology, neutron texture analysis provides insight into the deformation history of rocks.

Crystallographic texture is commonly described using pole figures, but the ultimate aim of texture analysis is to determine the ODF of crystallites in a polycrystalline aggregate in order to interpret this distribution in terms of the processes that produced it and to establish a relationship between the ODF and the macroscopic material properties. A pole figure is a stereographic projection that shows the distribution of a specific crystallographic direction (or plane normal or pole) relative to the sample reference frame. By measuring diffraction intensities from selected lattice planes, pole figures reveal the number of grains belonging to a particular crystallographic orientation, providing an experimental representation of preferred orientation densities for individual crystallographic poles. The ODF therefore provides a complete and quantitative description of the orientation distribution of all grains within the probed material. This then gives the probability of finding a crystallite with a given orientation in the polycrystalline aggregate, often expressed in multiples of a random distribution (m.r.d.), where a value of 1 m.r.d. corresponds to the texture-free state and deviations from this value indicate preferred orientations that lead to anisotropic mater­ial behaviour.

Time-of-flight (TOF) neutron diffraction for texture analysis has been implemented on several instruments at pulsed neutron sources, including SKAT (Dubna, Russian Federation) (Ullemeyer *et al.*, 1998 ▸), HIPPO (Los Alamos National Laboratory, USA) (Wenk *et al.*, 2003 ▸), GEM and ENGIN-X (ISIS Neutron and Muon Source, UK) (Kockelmann *et al.*, 2006*a* ▸; Malamud *et al.*, 2014 ▸), iMATERIA [Japan Proton Accelerator Research Complex (J-PARC), Japan] (Onuki *et al.*, 2016 ▸), and NOMAD (Spallation Neutron Source, USA) (Peterson *et al.*, 2021 ▸). TOF-based neutron texture analysis capabilities have also been demonstrated on the RIKEN accelerator-driven compact neutron source (RANS), enabled by the optimization of detector segmentation and background suppression (Xu *et al.*, 2020 ▸).

Many of these neutron diffractometers are designed to measure diffraction patterns using a polychromatic thermal or cold neutron beam, with a wide angular range provided by large detector arrays. Neutron energy and wavelength are straightforwardly measured using the TOF method, enabling a substantial portion of orientation space to be probed in a single measurement and significantly improving data collection efficiency. TOF texture analysis allows for multiple and overlapping diffraction peaks of multiphase materials to be analysed, enabling texture measurements under non-ambient conditions (Obasi *et al.*, 2012 ▸). Modelling of diffraction resolution effects has been used to extract additional microstructural information such as crystallite size in specific orientations (Wenk *et al.*, 2003 ▸; Kockelmann *et al.*, 2006*a* ▸) alongside ODF reconstructions using *EWIMV* in *MAUD* (Wenk *et al.*, 2010 ▸) or generalized harmonics in *GSAS* (Toby & Von Dreele, 2013 ▸) by Rietveld fitting multiple diffraction patterns simultaneously.

Recent advances in instrumentation and experimental methods have enabled spatially resolved neutron texture measurements using small gauge volumes for non-destructive studies of complex engineering components, cultural heritage objects, general geological materials and ceramics exhibiting texture gradients. This capability has been implemented on engineering neutron diffractometers such as ENGIN-X at the ISIS Neutron and Muon Source (Malamud *et al.*, 2014 ▸; Malamud *et al.*, 2016 ▸) and TAKUMI at J-PARC (Xu *et al.*, 2018 ▸; Xu *et al.*, 2024 ▸), with further developments on POLDI at PSI (Stuhr, 2001 ▸; Malamud *et al.*, 2025 ▸) and the future BEER at the European Spallation Source (Fenske *et al.*, 2016 ▸). These instruments enable spatially resolved measurements, allowing texture mapping across engineering components. Spatial resolution is achieved by restricting the incident neutron beam size and using radial collimators positioned between the sample and detector. Detectors are set up at 90°, to provide for all sample orientations a well defined gauge volume within the material, with ENGIN-X allowing experimental configurations which define cubic gauge volumes of 0.5, 1.0, 2.0, 3.0 and 4.0 mm side length. By scanning the sample relative to this gauge volume, localized texture information can be obtained.

Spatially resolved diffraction is particularly valuable for studying process-induced texture gradients in heterogeneous engineering materials, weld zones, surface-treated layers, additively manufactured structures and regions affected by mechanical deformation, as well as non-destructive characterization of composite and multi-material archaeological objects. Compared with dedicated texture diffractometers like HIPPO, engineering strain scanners such as ENGIN-X have reduced detector coverage. Consequently, more sample rotations are required to obtain sufficient pole-figure coverage. The use of small gauge volumes also requires precise sample alignment, multiple scan positions for component mapping and more advanced data processing strategies to handle the resulting datasets.

The present work focuses on the development of software tools to support spatially resolved neutron texture analysis, and we describe its implementation on the ENGIN-X diffractometer (Santisteban *et al.*, 2006 ▸). Texture information is required in many scientific and engineering disciplines, yet users are often not specialists in texture analysis. The pipeline presented here addresses the experimental and computational challenges associated with texture measurements of complex multi-part components. It provides an integrated workflow for experiment planning, evaluation of pole-figure coverage, prediction of directional absorption effects, processing of large datasets, application of corrections (*e.g.* normalization and absorption), and generation and visualization of experimental pole figures. The implementation builds on the previously developed and validated *NyRTeX* software (Malamud *et al.*, 2014 ▸) and extends this approach to be available and maintained within the *Mantid* framework (Arnold *et al.*, 2014 ▸).

The scientific value of this transition from *NyRTeX* (a standalone MATLAB routine, no longer actively maintained) to *Mantid* is multi-faceted. *Mantid* is a staple program of the neutron diffraction community, developed and maintained by a dedicated team of engineers, and its use is already integrated into the standard operational procedure of the majority of ISIS instruments. This provides a user with no administrative overhead involved with software installation at the point of conducting the experiment, as the instrument computers have the latest *Mantid* release pre-installed. For post-experiment analysis, *Mantid* is further available on the user *Data Analysis as a Service* instances by default, but for a personal computer *Mantid* fully supports Windows, Mac and Linux, and provides both conda installs and standalone packages. This paper details the development of software which will allow those running a texture experiment at ISIS to generate experimental pole figures, directly from the computer in the experiment hutch, as the data are collected. This provides an invaluable feedback loop as the experimental data are being collected and should facilitate better directed experiments, allowing for better data and better material insights.

We acknowledge that other texture analysis software packages exist and have broad applicability and user bases, and this work does not necessarily seek to replace these software packages. One such program is *MAUD* (Saville *et al.*, 2021 ▸). Depending on the situation and the analysis being done, it may be more appropriate to use an alternative program such as *MAUD* (as an example, *MAUD* has extensive native support for HIPPO at LANSCE, so it would probably not be advisable to use the present *Mantid* routine for processing data collected there). The advantage and value being provided by the creation of the new software arise from its natural integration into the workflows for the facilities already using *Mantid* (ISIS, ORNL, ILL, PSI). Additionally, as will be discussed in more detail below, *MAUD* performs whole pattern analysis, whereas the workflow presented here is intended to allow for fast model-free determination of texture at the point of data collection, without necessitating an adequate fit to entire patterns. A further advantage of *Mantid* being integrated into cross-facility workflows is that a user could learn to use the software in an experiment on ENGIN-X at ISIS and then use broadly the same routine for another experiment on POLDI at PSI (Malamud *et al.*, 2025 ▸) (with some adjustments to the pre-fitting steps to account for the difference in source types).

Furthermore, the remit of *Mantid* ends at the point of data reduction, pole-figure inversion and ODF reconstruction, and further quantitative analysis is not performed within the workflow. Instead, interfaces to established texture analysis packages such as *MTEX* (Bachmann *et al.*, 2010 ▸; Mainprice *et al.*, 2015 ▸) are provided, or else a user could choose to continue their data processing using another package such as *MAUD*.

The capabilities described within this article are implemented into the *Mantid* Version 6.15 release (Mantid Project, 2025 ▸).

Section 2 reviews spatially resolved neutron texture analysis on a strain scanner such as ENGIN-X. The Python processing pipeline and its individual components are introduced in Section 3, while Section 4 includes detailed implementation and operation steps in *Mantid*. In Section 5, the workflow is demonstrated using experimental data from a high-strength aluminium alloy measured on ENGIN-X at the ISIS Neutron and Muon Source. The same aluminium sample was previously investigated on this instrument and analysed using the earlier *NyRTeX* software, validating this methodology with respect to the texture results derived from X-ray techniques. Testing this implementation on the same sample allows comparison with these same X-ray results and agreement helps to validate this *Mantid* implementation. Finally, Section 6 provides a summary of the work and outlines perspectives for further development and applications of the workflow.

## 2. *NyRTeX*: spatially resolved texture analysis at a neutron strain scanner

ENGIN-X (Santisteban *et al.*, 2006 ▸) is a TOF neutron strain scanner, a white-beam diffractometer optimized to measure diffractograms at precise locations within bulk components along two perpendicular sample orientations.

**Figure 1:**
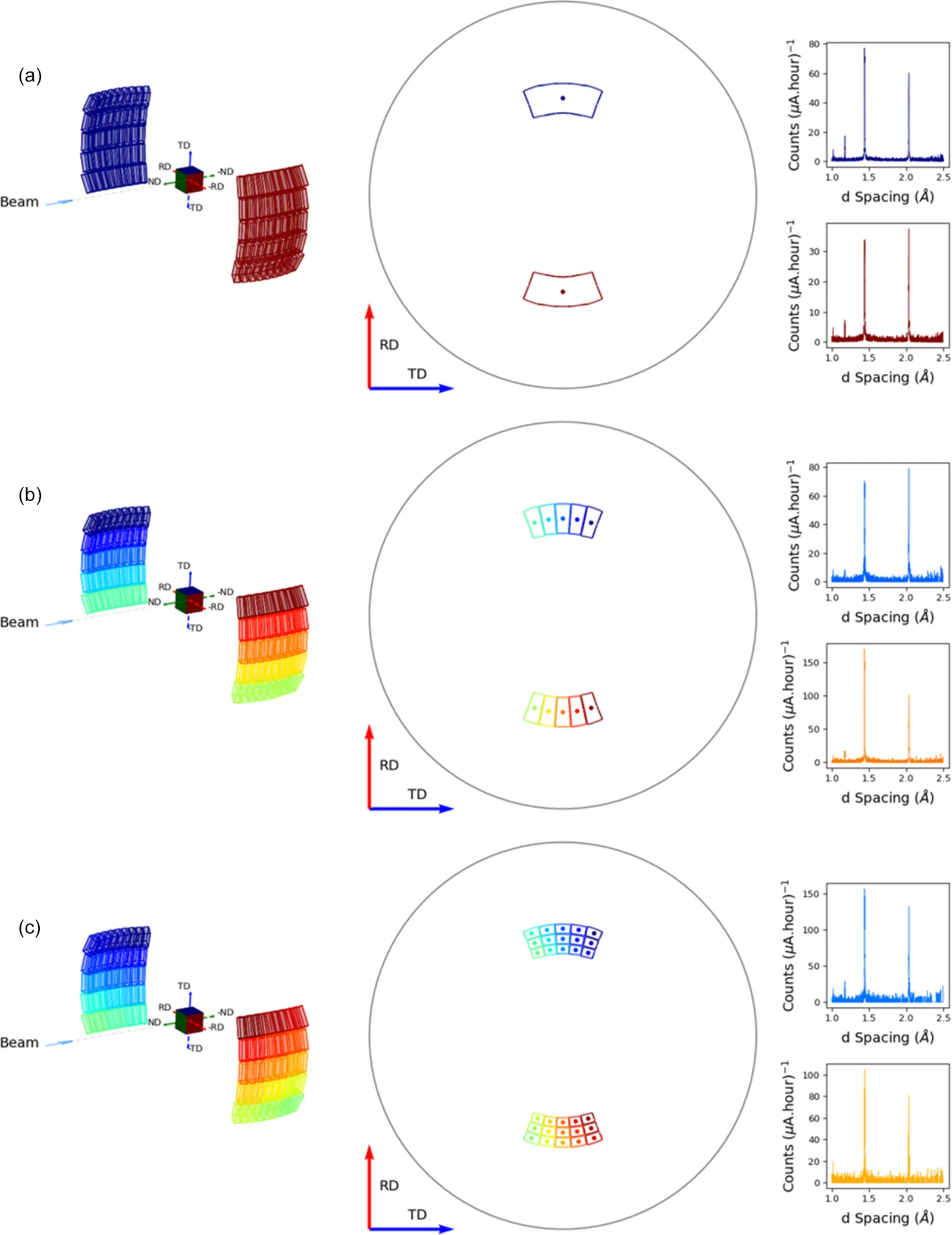
Depictions of the experimental geometry of a texture experiment on ENGIN-X. The first column shows a sample on a beamline with blocks of detector pixels arranged at ±90° to the beam direction. The sample directions are shown with red, green and blue coloured arrows (here labelled, for the sake of example, with some common labels, namely RD, ND and TD, being the rolling, normal and transverse directions, respectively, of a rolled metal sample). Groups of detectors can be combined to form virtual detectors that subtend a defined solid angle around the sample. The regions these virtual detectors occupy are displayed on the pole figures (central column), alongside the single point used to represent each virtual detector. The diffraction patterns associated with the virtual detector containing the central pixel of each bank are shown in the final column. Rows show different virtual detector groupings: (*a*) grouping all pixels in each bank, (*b*) grouping all pixels in each module and (*c*) grouping the pixels in each module into three subgroups.

As illustrated in Fig. 1 ▸, a polychromatic neutron beam travels along a curved guide and impacts the sample 50 m from the source. The scattered neutrons are collected by two detector banks positioned at ±90° to the incident beam, each covering ±16° horizontally and ±21° vertically, with the gauge volume defined by adjustable slits and radial collimators. Because the incident beam is polychromatic, a full diffractogram is recorded at a fixed scattering angle, providing information from multiple crystallographic families in a single acquisition. Each diffraction peak reflects the subset of crystallites whose plane normals are aligned with the scattering vector of the respective bank.

In standard strain scanning experiments, all detector elements within a bank are summed into a single diffractogram, sampling only two directions in pole-figure space simultaneously [Fig. 1 ▸(*a*)]. Texture analysis on such an instrument is made possible by subdividing each physical detector bank into smaller groups of detector elements, referred to as virtual detectors, each covering a reduced solid angle and therefore probing a distinct region of pole-figure space.

The ENGIN-X detector banks comprise five modules of nine detector blocks, each containing 26 vertical strip detector pixels. As shown in Figs. 1 ▸(*b*) and 1 ▸(*c*), grouping different sets of detector blocks changes the solid angle subtended by each virtual detector and hence the angular resolution of the resulting pole figures. In the figure, each block of detector pixels is shown as a sample-facing coloured cuboid, such that the centres of the detector faces fall on a spherical arc. For simplicity, the radial collimators have been omitted from the graphic, but these ensure each detector pixel can only detect signal from a controlled pre-defined portion of the illuminated sample. By combining data from multiple virtual detectors across a series of sample orientations, a set of incomplete experimental pole figures can be generated, from which the ODF is reconstructed using established inversion methods.

This approach was first implemented in *NyRTeX* (Malamud *et al.*, 2014 ▸), a MATLAB-based software package developed specifically for spatially resolved texture analysis on TOF neutron strain scanners. The present work extends this methodology within the *Mantid* framework (Arnold *et al.*, 2014 ▸), providing a Python-based pipeline with improved support for experiment planning, absorption correction and processing of large datasets from complex multi-part components.

## 3. Data reduction in *Mantid*

### 3.1. Overview

In general, the data reduction pipeline for texture analysis in a TOF diffractometer follows this broad structure:

(i) Loading raw data and unit conversion to *d* spacing.

(ii) Combining (‘focusing’) into virtual detector spectra and correcting.

(iii) Fitting diffraction peaks.

(iv) Compiling pole-figure data.

The specifics of achieving these steps can vary somewhat depending on the instrument and experiment, but typically combining and correcting the diffraction patterns will consist of calibrating the detector parameters, correcting the intensity collected in each detector to account for attenuation of the beam through the sample, and focusing the data for detector pixels or groups of detectors using the result of the calibration. Fitting the spectra is then performed, using *Mantid*’s fitting engine, to obtain optimal parameter values of the chosen peak function for all the peaks of interest in all the detector-grouped spectra. To compile this information into the tabulated pole-figure data, the spherical coordinates of each detector group relative to the specified intrinsic sample directions are calculated and appended into a table, along with one chosen peak function parameter value for that specific detector-grouped spectrum. In the next section (Section 3.2) we expand upon these steps further, discussing the approaches and considerations for each. Following this, in Section 4 we then cover the direct implementation of the pipeline, highlighting the mandatory and optional user inputs.

### 3.2. Conceptual pipeline

#### 3.2.1. Data combination and correction

**Figure 2:**
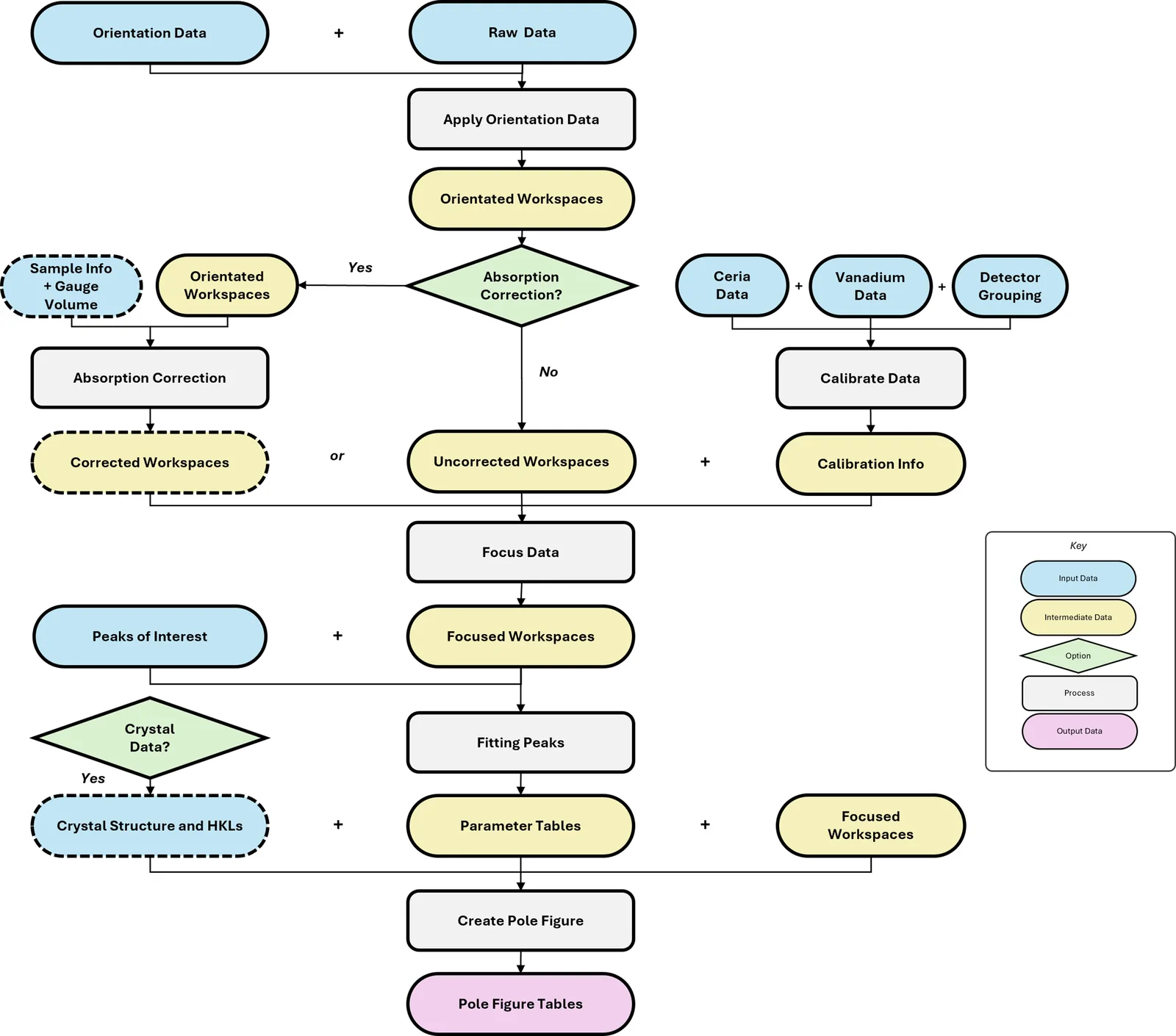
A schematic breakdown of the texture reduction pipeline. Blue nodes denote information or data which must be provided by the user. Yellow nodes denote intermediate data containers, usually in the form of *Mantid* workspaces. Grey nodes denote processes. Green nodes denote options for the user. The purple node denotes the output data. Dashed nodes denote data which may or may not be present/required, depending on the decisions made by the user.

The end target of the provided texture analysis pipeline is to output the data required for the construction of the pole figure *P*_*hkl*_(α, β). The approach is outlined in Fig. 2 ▸. This *P*_*hkl*_(α, β) compares the integrated intensity of a given *hkl* peak in the textured sample with the corresponding intensity for an equivalent sample with a perfectly isotropic distribution of crystallites. *P*_*hkl*_(α, β) is given as a continuous function of the angular coordinates α (latitude) and β (longitude).

In practical terms, a complete determination of this function cannot be achieved experimentally and so one must rely on the sampling of integrated intensities *I*_*hkl*_(α, β) for a discrete set of virtual detectors.

Further, any intensities determined experimentally will be a function not only of the desired sample texture but also of the sample and instrumental factors. Specifically, the additional effects that should be accounted for are beam attenuation, individual peak scattering power effects and instrumental effects specific to each virtual detector.

The attenuation of the neutron beam as it passes along the flight path through the sample will depend on the material the sample is made of, the extent to which the atoms within the material attenuate the beam and the length of the flight path through the material. The strength of this effect will be approximated using a Monte Carlo simulation, packaged into the correction factor *C*^abs^, where 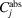 depends on the location of a specific detector *j*.

This correction removes the geometric and material effects of beam attenuation from the recorded intensity. Although attenuation correction can be applied after calibration and focusing (on the grouped spectra), this approach is less accurate and must be repeated if detector groupings are changed. Applying the correction at the raw per-pixel level preserves flexibility for later regrouping without loss of accuracy.

The attenuation correction is calculated on the basis of path lengths for scattered beams emanating from the *gauge volume*, the portion of the sample illuminated by the incident beam and visible to the detectors. The correction factor is computed via a Monte Carlo ray-tracing approach (Hamilton, 1957 ▸; Sears, 1992 ▸) where many scattering events are simulated within the gauge volume.

The Monte Carlo implementation of the attenuation correction has been benchmarked and validated within *Mantid* against the results for standard geometries of spheres (Dwiggins, 1975*b* ▸) and cylinders (Dwiggins, 1975*a* ▸). A summary of this validation is given in Figs. S1 and S2 in the supporting information.

In addition to the effect that sample attenuation has on the collected intensity, there will also be inherent variation in the intensity of detector pixels as a result of intrinsic factors of the instrumentation and setup. The factors to consider here are:

(i) variations in detection efficiency across different pixels,

(ii) variations in efficiency within a pixel across different neutron wavelengths,

(iii) variations in solid angle coverage from pixel to pixel,

(iv) variations in beam profile at different detector locations.

The combined effect of the instrumentation parameters on the measured flux from the sample is given by
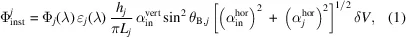
where θ_B,*j*_, *L*_*j*_ and *h*_*j*_ are, respectively, the mean Bragg angle, the distance to the sample and the height of the *j*th virtual detector, Φ_*j*_(λ) and ɛ_*j*_(λ) are, respectively, the incident neutron flux and detection efficiency at the wavelength λ, δ*V* is the gauge volume, *i.e.* the volume of the sample illuminated by the neutron beam that is effectively seen by the detector [as illustrated in Fig. 1 ▸(*a*)], 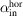 and 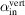 are the divergences of the incident beam in the diffraction plane and normal to it, respectively, and 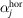 is the divergence of the scattered beam seen by the detector, as allowed by the radial collimator. While equation (1) is only explicitly correct for scattering in the equatorial plane, it has been assumed that the deviation from this, for out-of-plane detectors, will be small. Further, this equation makes the assumption that the quadrature sum of the contributions to divergence can be modelled as a Gaussian distribution.

In practice, this term is corrected for in a few separate parts. The solid angle correction and wavelength dependence terms, Φ_*j*_(λ)ɛ_*j*_(λ), are corrected with the vanadium normalization, V_norm_: 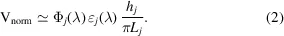
Vanadium is chosen as it acts, to a good approximation, as an incoherent isotropic scatterer, such that the intensity variations observed within the vanadium run are almost entirely due to these instrumental factors. The effect of the vanadium normalization can be seen, applied to the reference CeO_2_, in Fig. S9. This normalization reduces the spurious intensity variation resulting from the instrumentation factors [equation (2)].

The effects of beam divergence are collected and corrected as *C*^div^,
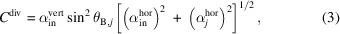
with the final component of equation (1), the gauge volume factor δ*V*, accounted for in the Monte Carlo calculated absorption correction, *C*^abs^.

The final effect which can be corrected for is the scattering power correction, 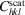, given by 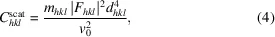
where *v*_0_ is the volume per atom, *F*_*hkl*_ is the structure factor (including the Debye–Waller factor), *m*_*hkl*_ is the multiplicity of the reflection and *d*_*hkl*_ is the interplanar distance of the particular reflection.

Together, this gives the final equation for the corrected intensity signal as 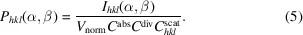


In addition to these corrections to intensity, the experimental data also need to be calibrated such that we can establish accurate values of DIFA, DIFC and TZERO – detector parameters which account for the spread of neutron energies, flight path lengths and offsets in the neutron timing electronics – and allow for the conversion of the collected data from TOF into *d* spacing or 2θ. This is done using a powdered ceria (CeO_2_) sample with well known and well defined Bragg peaks to determine the correct instrument parameters such that the conversion of the observed neutron TOF data into *d* spacing matches the expected values.

Examples of the quality of the fits of DIFA, DIFC and TZERO can be seen, with respect to the positional errors of the reference peaks, in Figs. S3–S7. These plots are produced as standard as part of the experimental workflow and allow the user to validate the quality of the unit conversion calibration.

The desire to combine detector pixels arises from the fact that, experimentally, the intensity of the diffracted signal collected within a given pixel will also contain noise. In order to improve the resolution of the meaningful crystallographic information above the experimental background, there are only three fundamental options: reduce the background to close to zero, subtract the background or maximize the diffraction signal. Instrument background on neutron diffractometers with radial collimators for strain is usually low; the diffraction pattern background is determined by non-Bragg scattering, including incoherent and inelastic scattering.

Assuming the experimental setup is already optimized for minimizing noise, one approach is to increase the signal, with the following main strategies: run the experiment for longer, aggregate detector pixels or increase the gauge volume. Running the experiment for longer, while often preferable, is not always practical under the constraints of experimental time allocation and beam availability at neutron sources and can additionally introduce complications surrounding sample irradiation.

The alternative, detector aggregation, is an established approach (Kockelmann *et al.*, 2006*a* ▸; Malamud *et al.*, 2014 ▸; Xu *et al.*, 2018 ▸) and can often be a suitable option, sacrificing some angular (texture) resolution for effectively a single detector with increased solid angle coverage and a proportional increase in signal, or by sacrificing spatial resolution by selecting a larger gauge volume.

In practice, a real experiment requires consideration of all factors, with experimental collection times set to be as long as practical and the gauge volume set as large as is it can be to achieve the desired spatial resolution, and then the detector grouping can be selected after the fact to achieve sufficiently high-quality spectra for fitting.

As alluded to, the primary advantage of virtually grouping detectors is the flexibility – it can be done in software after experimental data collection and tweaked to provide the ideal balance of signal-to-noise ratio and angular resolution. Performing these detector signal agglomerations in practice requires care to ensure that each individual pixel spectrum is correctly assigned to the correct group, but our approach in *Mantid* provides both standard virtual-detector griddings (full bank, 2 × 2 × 5 and 2 × 3 × 5) that can be used straightaway and extensive support for creating and using custom detector pixel combinations. There is support for groupings based on the instrument hierarchy, *i.e.* pixels are grouped into modules and modules into banks, and there are algorithms which allow a user to split the detector pixels into *N* groups under a given component layer (*e.g.* group the pixels under each module into nine separate groups). There is also support for creating groups based on scattering angle and there are instrument visualization tools to verify that the groupings have been defined as expected.

#### 3.2.2. Bragg peak fitting

Once the data have been corrected, calibrated, focused and suitably aggregated, the next requirement is to extract the crystallographic information of interest from the resulting spectrum. This typically means identifying the Bragg reflections of interest and fitting these peaks with parameterized model functions. By optimizing the model parameters to achieve the best fit to each peak in each spectrum, the variation in peak parameters across the datasets can be used to indicate the evolution of crystallographic factors as a function of detector position. Two examples here are fitting the peaks with functions that offer a measure of peak area (intensity) and peak centre, and tracking these parameters as indicators of the alignment of the relevant crystal plane with the scattering vector and crystallographic strain, respectively. The quality of the conclusions that can be drawn from this approach is tied to the quality of the fit of the model function to the experimental data – a function with few parameters providing a close match to the experimental data points is a much more reliable measure of the underlying crystallography than a function with many parameters that does not align well with observation.

This individual peak fitting approach is different from full profile approaches used by other software packages such as *MAUD* (Saville *et al.*, 2021 ▸) which simultaneously refines crystal structure, phase fraction, microstructure and instrument parameters alongside texture parameters, using approaches such as WIMV and spherical harmonics for ODF reconstruction. These full profile approaches offer a convenient way of reducing some of the parameter redundancy discussed above by tying all profile properties related to the underlying crystal model together. Such approaches are well suited to low-symmetry multiphase systems with significant diffraction peak overlap. However, these capabilities being effective can require much more substantial user expertise, as the quality of the result depends heavily on the quality of the underlying model and the execution of the refinement.

An individual fitting approach is much more flexible, especially at the point of initial experimental data collection, as a pole figure can be produced for any experimentally observed Bragg peak without requiring the definition of a crystal model. The *Mantid* workflow does allow for providing the crystal model and this is required for providing index pole figures, but in principle it is not required at the outset. This could prove particularly useful for investigation of the nature and texture of impurity phases which have not been well characterized *a priori*. *Mantid* additionally does currently provide some support for performing Pawley fits, functionality which is intended to be extended to this texture workflow and which would provide a middle ground option between the individual peak fitting and performing a full profile refinement with external software.

#### 3.2.3. Pole-figure data compilation

Finally, with the relevant information extracted from the spectrum, the last step in the reduction is to compile this information with the detector groups’ positional information. As the goal of this reduction workflow is to identify the change in crystallographic properties in different directions around the sample, it is necessary to know the position of each detector relative to these pre-defined sample directions for each spectrum. These positions are typically given as spherical coordinates, with in-plane ‘azimuthal angles’ and out-of-plane ‘zenith angles’. These positional angles are then combined with a desired fitting parameter for a single Bragg peak to form the final reduced data table. Again, reiterating the goal of extracting information on the positional dependence of crystallographic data, it is often the case that, in order to achieve adequate positional sampling around the specimen, multiple experimental runs are aggregated into a single reduction table. Here, each run orients the sample in a different configuration, effectively repositioning the detectors relative to the intrinsic sample axes and increasing the directional sampling around the sample.

## 4. Implementation and operation

### 4.1. Overview

Within this section we will cover the basic steps and considerations of the texture reduction workflow, as implemented in *Mantid* Version 6.15. We encourage the reader to refer to the *Mantid* documentation (links below) for additional information on operating procedure, particularly as the implementation is improved and refined.

It is convenient to assume that the reader has some basic understanding of *Mantid* functionality (Arnold *et al.*, 2014 ▸), but as a minimal reference the main concepts to be familiar with are *Mantid* ‘workspaces’ and ‘algorithms’.

Within *Mantid* the primary containers for data are ‘workspace’ objects. In the context of this workflow these workspaces contain either a table of data, with rows and columns of data much like an spreadsheet, or neutron histogram data, made up of different spectra and their collection bins, intensities and errors. As well as holding direct experimental data, these workspaces also contain useful additional information such as experimental logs, sample information, history of transformation operations (algorithm history) and more. For the purpose of understanding this data reduction workflow, one simply needs to understand that throughout the pipeline the data being reduced will be held and operated upon within the wrappings of these workspaces.

The second concept to have an awareness of is *Mantid* algorithms. These are just predefined methods which take input workspaces and sets of input parameters and perform an operation upon the workspace to return new or updated workspaces. Additionally, as mentioned previously, each workspace contains an algorithm history, which offers clarity and reproducibility to the final results.

### 4.2. Absorption correction

Typically, the first stage in the reduction pipeline is to correct the recorded neutron intensities for attenuation of the incident and scattered beams as they pass through the sample.

To perform absorption correction, the user must supply:

(i) The raw data files (.nxs format) for each experimental run to be corrected.

(ii) A *reference workspace* defining the sample geometry and material in its reference orientation (typically as mounted on the beamline).

(iii) The sample shape, provided as:

(*a*) an XML string describing the geometry using constructive solid geometry (CSG) or

(*b*) an STL file.

(iv) The sample material definition, specified via *Mantid*’s SetSampleMaterial algorithm.

(v) The sample orientation for each run, provided via a single orientation_file which contains either:

(*a*) Euler angles for each goniometer axis, with axis alignment and rotation sense defined by euler_scheme and euler_axes_sense or

(*b*) flattened rotation matrices for more complex positioning systems (*e.g.* ENGIN-X Cybaman).

The reference workspace holds the full definition of the sample in its reference position. For each run, the orientation from the orientation_file is applied to the reference shape, updating the run’s workspace *Goniometer* object. This approach ensures that the material definition and base geometry are set only once and then transformed for each experimental configuration.

Since the absorption correction factor depends on the beamline geometry and collimation, the user provides an *illumination volume* (beam footprint and detector visibility without considering the sample), either by selecting a preset for standard configurations or by supplying a custom XML definition. Internally, the software rasterizes the illumination volume and discards illuminated voxels (volumetric pixels) which lie outside the sample shape to obtain the gauge volume.

Optional parameters (*e.g.* number of rays, random seed) can be passed in a Python dictionary to adjust the Monte Carlo behaviour.

The algorithm can be run to return the calculated attenuation at a given *d* spacing or TOF without applying the correction itself, aiding experimental planning. An optional beam divergence correction is also available, implemented as in *NyRTeX* (Malamud *et al.*, 2014 ▸) [given in equation (3) in that paper, equation (1) here], requiring user-specified in-plane and out-of-plane divergence values for the incident beam and in-plane divergence for the detectors.

The output of this stage is a set of per-pixel attenuation-corrected workspaces for each run. These can be focused into arbitrary groupings in later stages without needing to repeat the correction.

**Figure 3:**
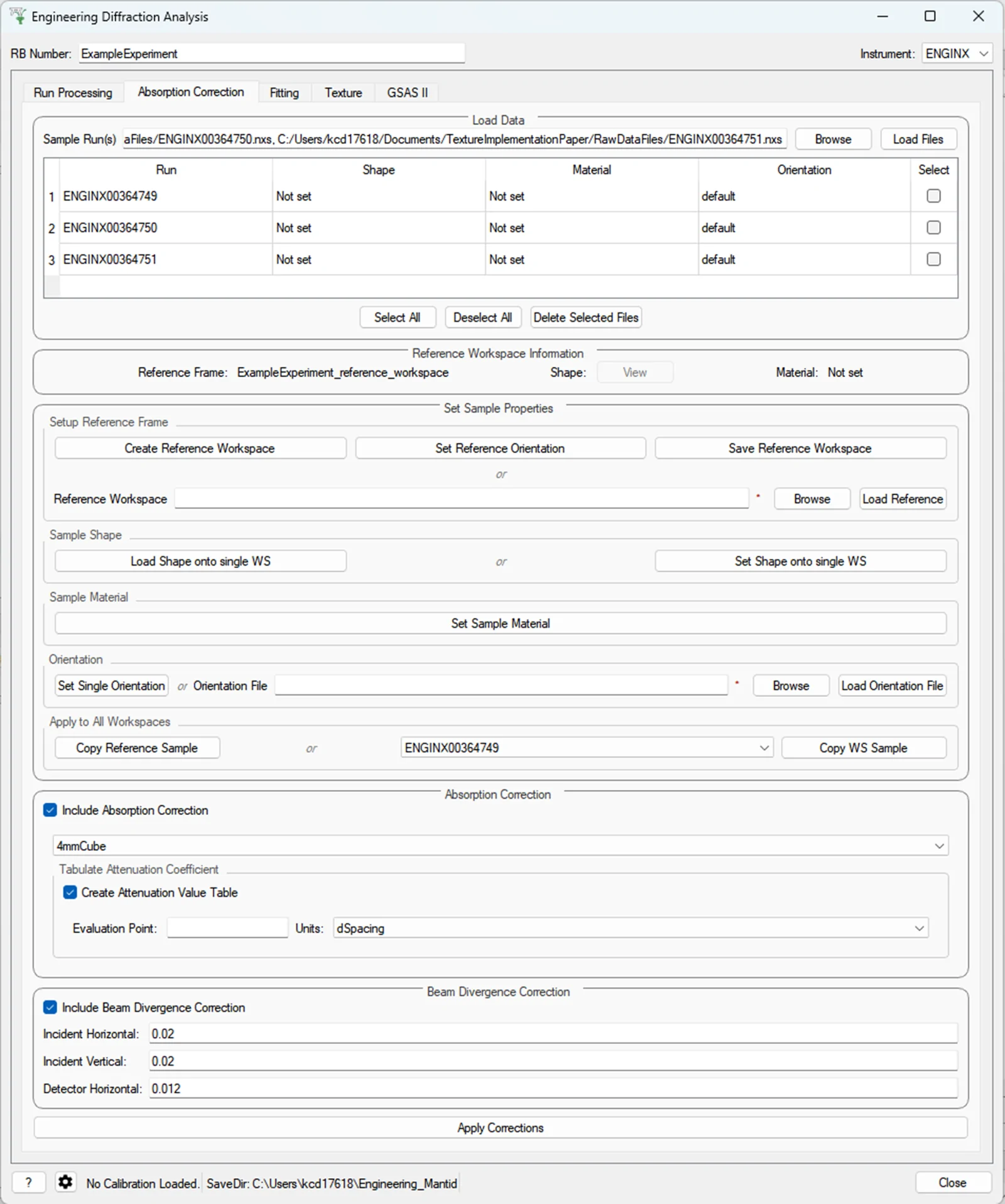
User interface for absorption correction. The design is intended so that the user works their way down from the top to the bottom of the interface.

Fig. 3 ▸ shows the user interface provided for performing the procedures outlined above. Navigation of the tab is designed to be run from top to bottom and is aided by hover tool tips explaining each option.

The top section of the interface allows loading all the relevant files (Browse and Load Files). From here the user next sets up the reference workspace (Create Reference Workspace) with a neutral (Set Reference Orientation) sample shape (from an STL file with Load Shape and from a CSG with Set Shape) and defining the sample material (Set Sample Material).

The orientations on each run can either be set individually on loaded runs (Set Single Orientation) or loaded from file (Browse and Load Orientation File).

Once this is done the reference sample can be copied to all the runs (Copy Reference Sample).

This completes the workspace setup. The absorption correction options are included in Fig. 3 ▸, with the bottom left-hand cog icon leading to more settings. The experimental gauge volume is set here, as well as the option to create a table of attenuation coefficients for each detector group at a specified evaluation point.

### 4.3. Calibration and focusing

Following attenuation correction (if applied), the next stage is to calibrate the detector parameters and focus the per-pixel data into user-defined groupings. This stage also applies the vanadium normalization to remove detector efficiency variations and to account for the wavelength dependence of the neutron flux profile.

The calibration step determines the instrument-specific parameters DIFA, DIFC and TZERO (Toby & Von Dreele, 2013 ▸), which convert the TOF to more crystallographically relevant units (*e.g. d* spacing or 2θ) and account for:

(i) change in neutron wavelength distribution (DIFA), *e.g.* due to absorption,

(ii) total flight path and detector 2θ angle (DIFC),

(iii) electronic timing offsets and sample positioning errors (TZERO).

The focusing step then aggregates data from multiple pixels into a higher-statistics spectrum, and vanadium normalization corrects for pixel-to-pixel efficiency variations and normalizes to the incident beam profile.

The calibration requires:

(i) Raw or attenuation-corrected .nxs files for the experimental runs.

(ii) A calibration run for the current beamline cycle, *e.g.* with ceria (CeO_2_) powder, to calibrate the flight paths and 2θ angles of virtual detectors.

(iii) A pre-calculated full-instrument calibration file for initial parameter estimates.

(iv) The desired detector grouping, provided from one of the following sources:

(*a*) loaded from one of the pre-defined detector groupings (*e.g.* ENGINX: North Bank, South Bank, Both Banks, Texture20, Texture30),

(*b*) loaded from a custom grouping file (.cal or .xml),

(*c*) provided as an inline group definition specifying detector IDs.

The procedure for calibrating the detector parameters is then as follows:

(1) Load the pre-calculated full-instrument calibration to initialize DIFA, DIFC and TZERO.

(2) Convert the ceria data to *d*-spacing units using these initial parameters.

(3) Identify and fit the known Bragg reflections of ceria using *Mantid*’s PDCalibration algorithm.

(4) Optimize DIFA, DIFC and TZERO for each detector group so that the observed peak positions align with the literature ceria *d* spacings.

Once the calibration parameters for all detector groups have been calculated, the experimental runs are then focused in the following manner:

(1) Using the calibrated parameters, group the vanadium run data into the selected detector groupings.

(2) Smooth and spline-fit the grouped vanadium spectra to produce continuous correction curves.

(3) For each experimental run, convert the spectrum to *d* spacing, group pixels using the calibrated parameters and divide the diffraction patterns by the vanadium correction curves.

The result is a set of calibrated, focused and efficiency-corrected diffraction patterns for each run and for the specified groupings, ready for peak fitting.

**Figure 4:**
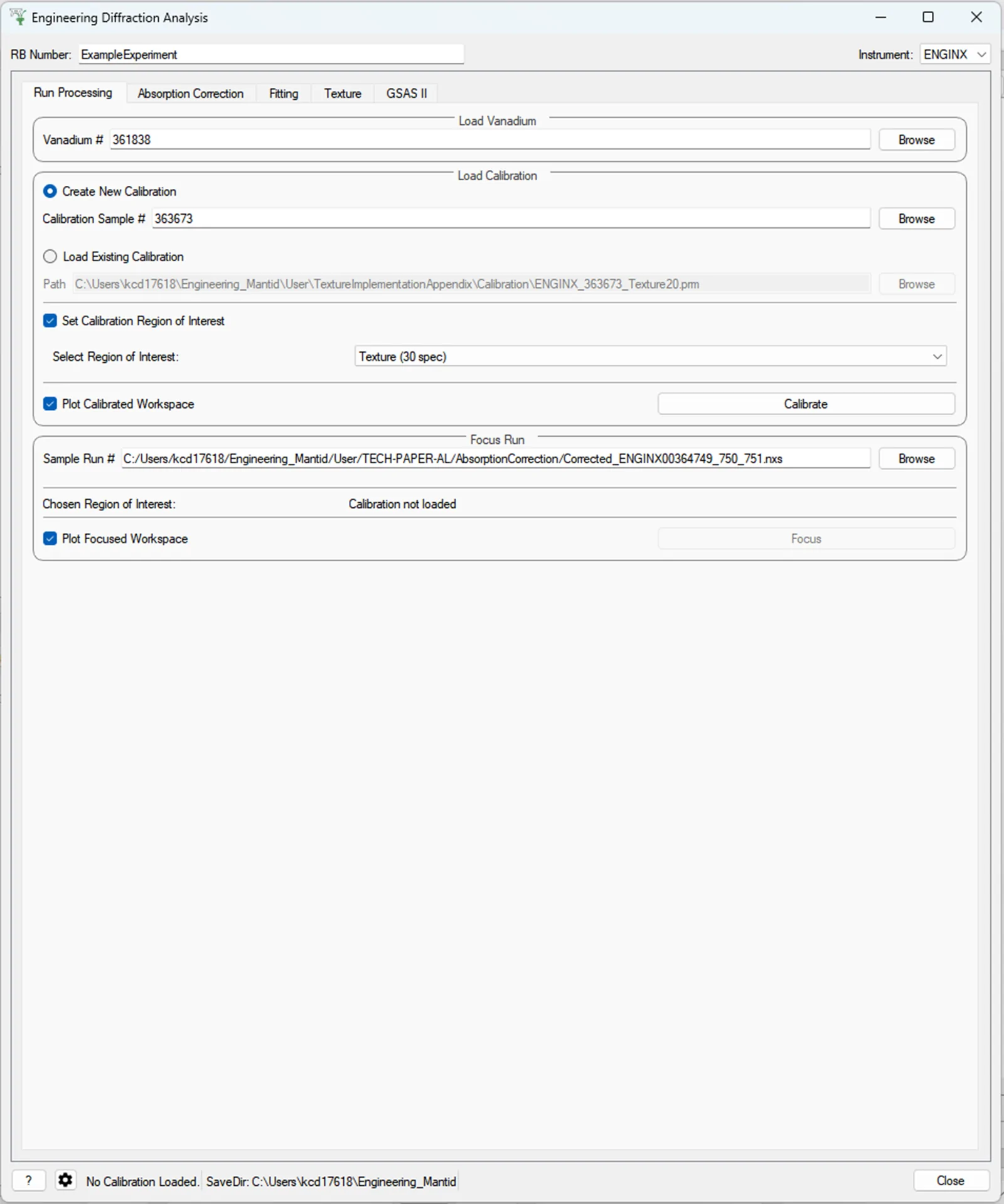
User interface for calibration and focusing. The interface design intends users to work from top to bottom.

Fig. 4 ▸ shows the user interface provided for performing the procedures outlined above. The vanadium sample is set in Vanadium # and the CeO_2_ is set in Calibration Sample #, with the virtual detector grouping given in the Select Region of Interest drop down box (including an option for supplying custom definitions). Sample Run # is used to specify the data sets for calibration and focusing operations.

### 4.4. Fitting

Once the experimental data have been corrected, calibrated, focused and grouped, the next step is to extract quantitative crystallographic information from the resulting spectra by fitting the Bragg peaks of interest. For texture analysis, the primary peak parameters of interest are the integrated intensities (areas) of diffraction peaks. This step is performed using *Mantid*’s *Fitting Engine*, with the aim of requiring minimal user intervention while maintaining flexibility in the choice of peak models and fitting strategy. As a result of on-going development, the implementation discussed here reflects the current state of the automated peak fitting script at the time of publication. The user interface also offers fitting support, offering full access to the *Mantid Fitting Engine*, but this approach is more user-involved and so will not be discussed in depth (more information on performing manual data fits is readily available in the *Mantid* documentation).

The fitting process is carried out in two stages: an *initial fit* to provide stable starting values for the fit parameters, followed by a *refined fit* to optimize the parameter values for each detector grouping individually.

For each experimental run and corresponding data set for a given sample angle and gauge volume position and for each user-defined peak of interest, the user specifies:

(i) the functional form of the peak (by default for neutron diffraction lineshapes, a BackToBackExponential),

(ii) the functional form of the background (by default, a LinearBackground),

(iii) the approximate *d*-spacing position of the peak,

(iv) the width of the fitting window around the expected peak position (typically ∼ 0.1 Å).

The default functions (LinearBackground + Back­To­Back­Exponential) are chosen to be appropriate for neutron diffraction data from instruments such as ENGIN-X and the default parameters are typically set with *d*-spacing dependence, but the user may provide alternative peak/background models and/or default parameters, if required.

In the initial fit stage, the selected peak shape is fitted to each peak in a summed spectrum across all detector groups. Certain parameters are then constrained as to how much they can vary from these initial values for subsequent fits across the individual groups. This is especially useful for fitting peaks with poor statistics in an individual detector group due to experimental setup/inherent sample texture. This is because for these types of fit, without appropriate constraining, the fit might preferentially fit random features in the noise – the constraints impose the condition upon noisy data that any peak that is to be fitted should have approximately the same position and shape parameters as the global fit. By default, the *A* and *B* parameters of the exponential tails in the BackToBackExponential function are also tied in subsequent fits, as these are primarily governed by the instrument resolution and any group-specific dependence is also sample dependent.

After the initial fit, subsequent fits can be performed on a series of smoothed transformations of the detector grouped data and/or the grouped data themselves. By default, the code uses a series of smoothed data, where the histograms are rebinned to combine every three bins, followed by every two bins. This smoothing provides a trade off of reducing the influence of statistical and background noise on an individual fit but affecting the peak shape and so, by extension, the fit values of shape parameters such as *A*, *B* and *X*0. By providing a series, it is intended that for each subsequent fit the starting values of the peak parameters are close enough to the optimal solution that the effect of smoothing on peak shape is accounted for. The primary parameters of interest from an engineering point of view are the intensity and the peak position. This dual fit allows parameters to be appropriately fitted to poor-statistic peaks, without being erroneously fitted to artificial features in the background. Between each fitting step the code evaluates the signal significance for each spectrum and peak using the metric *I*/σ_*I*_, where *I* is the fitted peak intensity (integrated area) and σ_*I*_ is its fitted uncertainty from the covariance matrix. This ratio provides a quantitative measure of whether the fitted peak represents a statistically meaningful Bragg reflection. The threshold for this significance test is user-configurable; by default, only peaks with *I*/σ_*I*_ exceeding this threshold are considered valid.

For spectra that fail the *I*/σ_*I*_ peak fit test, the peak is marked as invalid and the chosen parameter is set to a fallback value, which can be one of the following:

(i) 0.0,

(ii) a user-specified parameter-specific default,

(iii) NaN (to indicate no valid measurement) or

(iv) a statistical aggregate (minimum, mean or maximum) of the corresponding parameter across all valid spectra for that run.

Users can choose between these different options, which primarily function to allow flexibility for exporting fitting results to various analysis software packages. Setting to zero can be appropriate for some parameters (*e.g.* failed fits reporting zero intensity) but is misleading for a parameter such as peak position. However, depending on which analysis software the user intends to use to process the results, zeros may be easier to handle than NaN.

Additionally, the selection of specific options will modify the appearance of the experimental pole-figure plots which are generated and may make these plots easier to understand at a glance. Setting the unfitted values to zero allows an intensity plot to flag up strongly textured peaks *versus* where no peak intensity is returned, but using NaN results in points with no peak intensity being omitted from the plot. For some of the other parameters, it might be telling to see how the fit results deviate from an average value (above or below). Therefore, assigning these values to failed fits is useful.

Once all peaks for all detector groups and experimental runs (all goniometer angles) have been processed, the results are collated into *Mantid*TableWorkspace objects. One table is produced for each fitted peak in each experimental run. Each table contains:

(i) the spectrum index (identifying the detector grouping),

(ii) the fitted parameter values for that peak (*e.g.* peak centre, integrated intensity, width),

(iii) the estimated uncertainties of each fitted parameter.

These tables form the core numerical output of the fitting stage and serve as the input to the pole-figure compilation stage (Section 4.5), where the spatial dependence of the selected parameter(s) is analysed in the sample reference frame.

### 4.5. Compiling pole-figure data

The final stage of the pipeline transforms the fitted peak parameters into a spatially resolved dataset for texture analysis, mapping the measured values onto the sample’s crystallographic reference frame.

To run this stage, the following inputs are required from the user:

(i) the focused workspaces for each run,

(ii) the corresponding fit parameter tables for the selected peaks and

(iii) the intrinsic sample directions in the laboratory frame.

Additionally, the user can provide some optional parameters including the corresponding *hkl* indices for each peak, the sample crystal structure (which itself is provided via a .cif file or by explicitly providing the lattice, space group and atomic basis) and a *d*-spacing tolerance for excluding spurious peaks from the final table.

Once these inputs are available, the detector group positions are transformed into the sample reference frame and expressed in spherical coordinates, using the following operations:

(1) For each detector group, read its position vector from the focused workspace in the laboratory frame.

(2) Apply the goniometer transformation and intrinsic axis transformation to convert to the sample reference frame.

(3) Convert the resulting vectors to spherical coordinates, where:

(*a*) azimuthal angle in the plane is defined by the first and last sample axes and

(*b*) zenith angle is measured from the second sample axis.

Additional corrections or filter operations can then be applied to refine the dataset:

(i) If *hkl* values and a crystal structure are provided, apply a scattering power correction [*NyRTeX*, equation (2)] to account for multiplicity and structure factor effects.

(ii) If *d*-spacing filtering is enabled, remove values outside the specified tolerance of:

(*a*) the expected *d* spacing for the given *hkl* or

(*b*) the run-average *d* spacing if *hkl* is not provided.

The results from each run are then compiled into tables of pole-figure-ready data, where each table contains the detector grouping position in spherical coordinates and the selected fitting parameter value (*e.g.* intensity, peak centre). The final step in the pipeline is to take the sets of tables generated for different experimental runs and the same peak and combine all the data into single output tables, which can be exported to texture analysis packages such as *MTEX*.

### 4.6. Pole-figure visualization

Alongside the fundamental data reduction pipeline, an additional visualization capability has been developed which allows the user to visualize the pole-figure data generated from the pipeline. There are two main view options, scatter or contour, with two types of projection available, stereographic or azimuthal. The projection options offer different ways of viewing the two angular components of the spherical coordinate representation of detector positions in a two-dimensional pole figure. The viewing options then allow the user either to see the individual rows in the pole-figure table as individual points on the pole figure, where the colour of the point corresponds to the parameter value, or to interpolate the full two-dimensional pole figure using the experimental values from the table and view this as a continuous scatter plot.

Examples of these visualizations can be seen in the figures discussed in later sections (*e.g.* Figs. 8 and 10)

**Figure 5:**
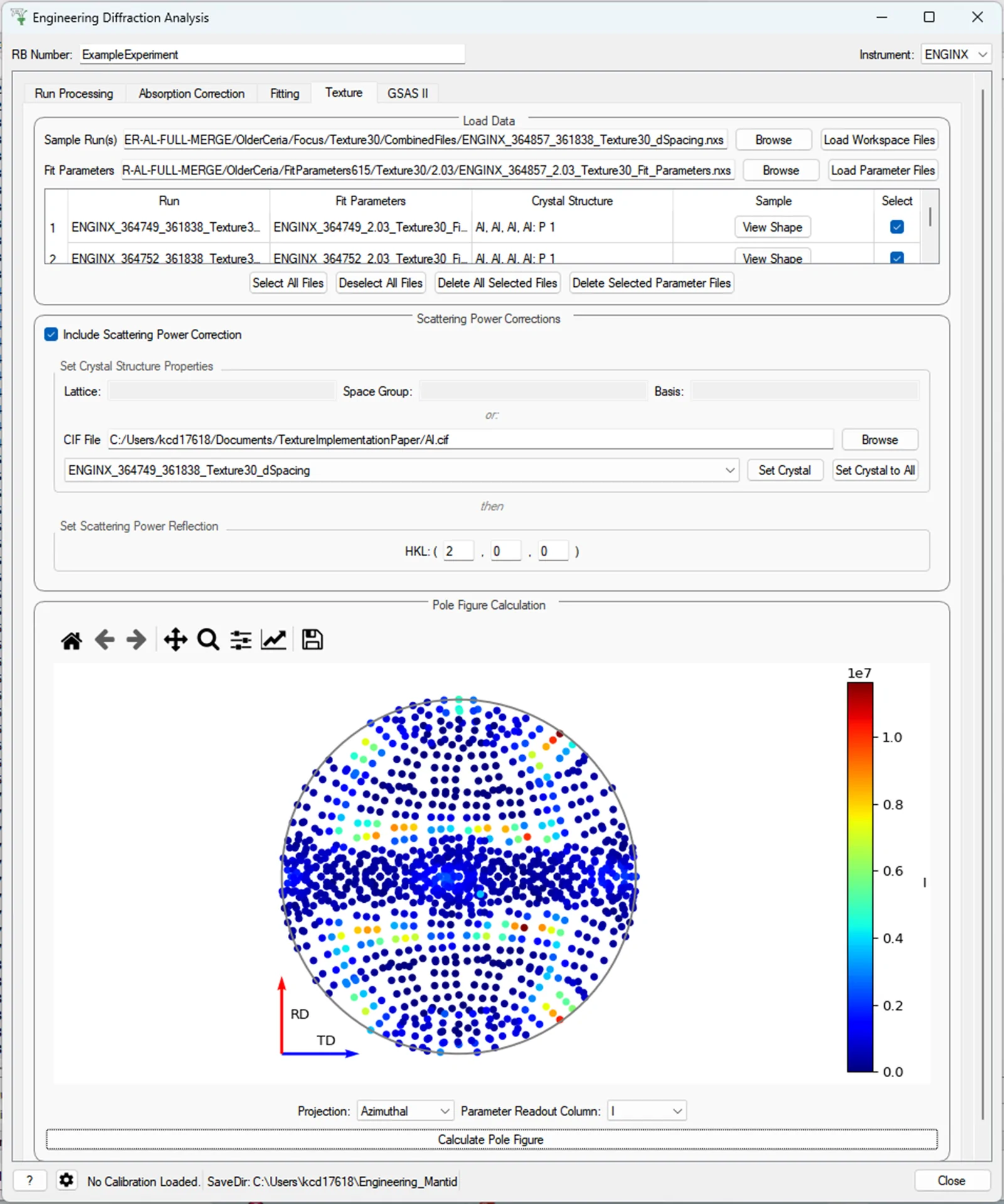
User interface for pole-figure compilation, designed to guide the user from top to bottom.

Fig. 5 ▸ shows the user interface provided for performing the procedures outlined above. The focused sample runs are loaded with Browse/Load Workspace Files and the fit­ting parameters are loaded with Browse/Load Parameter Files. The crystal structure can then be defined with the Set Crystal Structure Properties section (defined either with a .cif file or by providing the individual elements such as lattice parameters, space group and crystal basis unit). The *hkl* can then be provided and the projection and plot parameters selected. Further options are available in the settings menu (cog symbol, bottom left).

## 5. Experimental validation of the pipeline

### 5.1. Al sample and experiment parameters

To validate the pipeline, the same high-strength aluminium specimen from *NyRTeX* (Malamud *et al.*, 2014 ▸) was characterized using the new *Mantid* routine. The *NyRTeX* method­ology was validated extensively using this sample (Malamud *et al.*, 2014 ▸). Reproducing the results of the *NyRTeX* study therefore provides confidence that both the shared [equation (1)] and newly implemented routines – Monte Carlo attenuation correction, ceria calibration and peak fitting – behave reliably; the per-step validations in the supporting information support this at the level of the individual algorithms.

**Figure 6:**
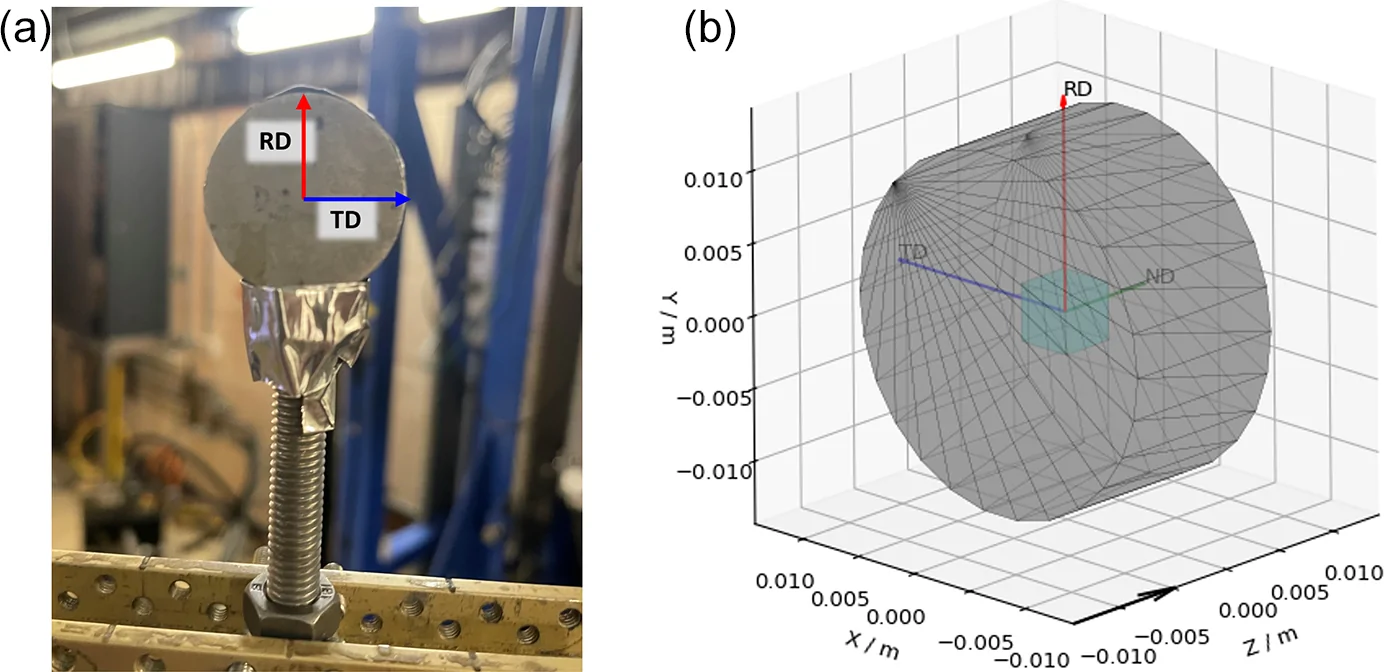
(*a*) A photograph of the sample mounted on the beamline. (*b*) A screenshot of the sample geometry, intrinsic directions and gauge volume defined within *Mantid*. The labels are RD (rolling direction) in red, ND (normal direction) in green and TD (transverse direction) in blue. These are standard sample directions for a rolled-sheet sample. The experimental gauge volume is shown in the *Mantid* screenshot as a light-blue cube, while the beam comes along the +*z* direction from a source at −*z* (shown by the black arrow on the *z* axis).

The aluminium sample consisted of a 12.6 mm thick plate of AA7150 (86.28 wt% Al, 6.9 wt% Zn, 2.7 wt% Mg, 2.5 wt% Cu, 1.5 wt% Fe, 0.12 wt% Si) in the T6 condition. To produce approximately homogeneous specimens, five slices corresponding to different depths were cut by electro-discharge machining (EDM) from a 55 × 92 mm plate. Six 25 mm diameter discs were separated by EDM from each slice and stacked with cyanoacrylate adhesive, producing a cylindrical specimen of 12 mm in height with a homogeneous microstructure (Malamud *et al.*, 2014 ▸). The sample directions are denoted RD (rolling direction), ND (normal direction) and TD (transverse direction), the standard labels for a rolled sheet. The specimen corresponding to the mid-thickness of the original plate was measured on ENGIN-X and an image of the initial orientation of the sample and the *Mantid* definition can be seen in Fig. 6 ▸. TOF diffractograms were collected for 37 specimen orientations, using a gauge volume of 4 × 4 × 4 mm and an incident beam divergence of 0.4° × 0.5°, and by employing an Euler goniometer for sample rotation. For each sample orientation, three acquisitions were measured with counting times of 5 min per acquisition, which allows for effective production of 5, 10 and 15 min representative datasets.

### 5.2. Experimental pole-figure creation

**Figure 7:**
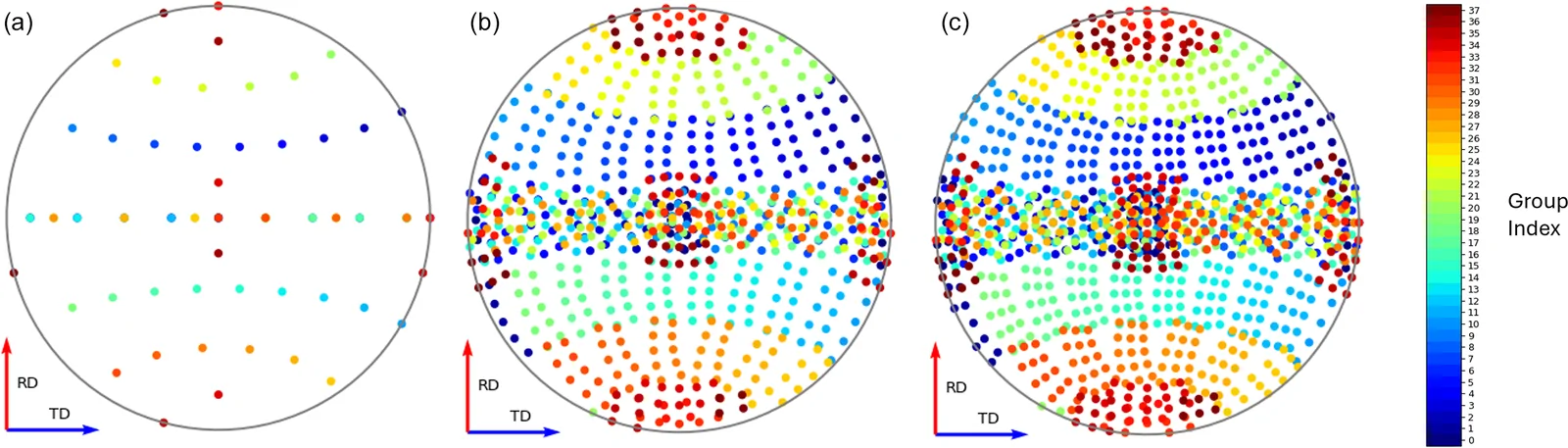
The experimental pole-figure coverage of the experiment for detector groups (*a*) by bank (2 × 1 × 1), (*b*) by splitting each bank into 2 × 2 × 5 groups and (*c*) by splitting each bank into 2 × 3 × 5 groups.

These data sets were processed using three different detector grouping schemes: grouping all the pixels in the bank (2 × 1 × 1), employing a 2 × 2 × 5 gridding by subdividing each bank into two horizontally (diffraction plane) and five vertically, and a 2 × 3 × 5 gridding, which leads to a pole-figure angular coverage for each run of ∼16° × 40°, ∼8° × 8° and ∼5° × 8°, respectively. The pole-figure coverage obtained by employing the different gridding schemes is presented in Fig. 7 ▸, with the goniometer angles for each run available in the supporting information (Table S1).

**Figure 8:**
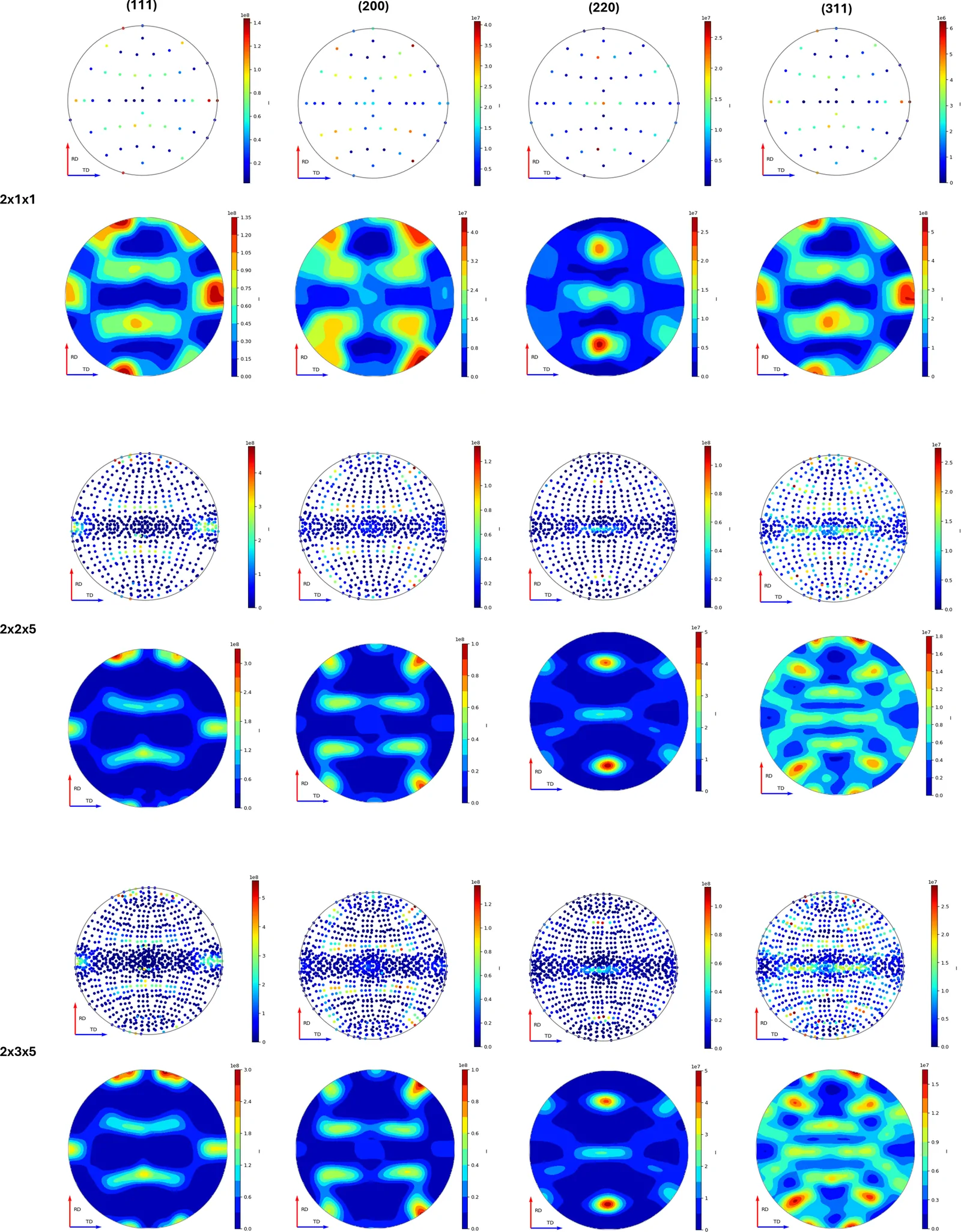
Experimental pole figures for intensities (given by colour bars) of four Bragg peaks of the 5 min collection aluminium dataset, shown as both experimental points and interpolated contour plots, for each of the detector grouping schemes.

After fitting, the experimental pole figures were created. For each grid scheme, the experimental pole figures (111), (200), (220) and (311) for the 5 min dataset are shown in Fig. 8 ▸, together with the corresponding contour maps created by the *Mantid* visualization tools. It can be seen that consistent textures are implied by the groupings of 2 × 2 × 5 and 2 × 3 × 5, and reasonable approximations to the same texture are made by the far lower spatial resolution of the bank-wide grouping. In the supporting information, Fig. S8 shows the 2 × 3 × 5 grouping for the (311) reflection and highlights that some of the strongly textured regions contain points sampled from different virtual detector modules. As such, the raw data in these regions have many of the sources of intensity variation discussed in Section 3.2.1. The presence of coherent texture results in these regions is consistent with the applied corrections successfully accounting for these variations.

### 5.3. Orientation distribution function

**Figure 9:**
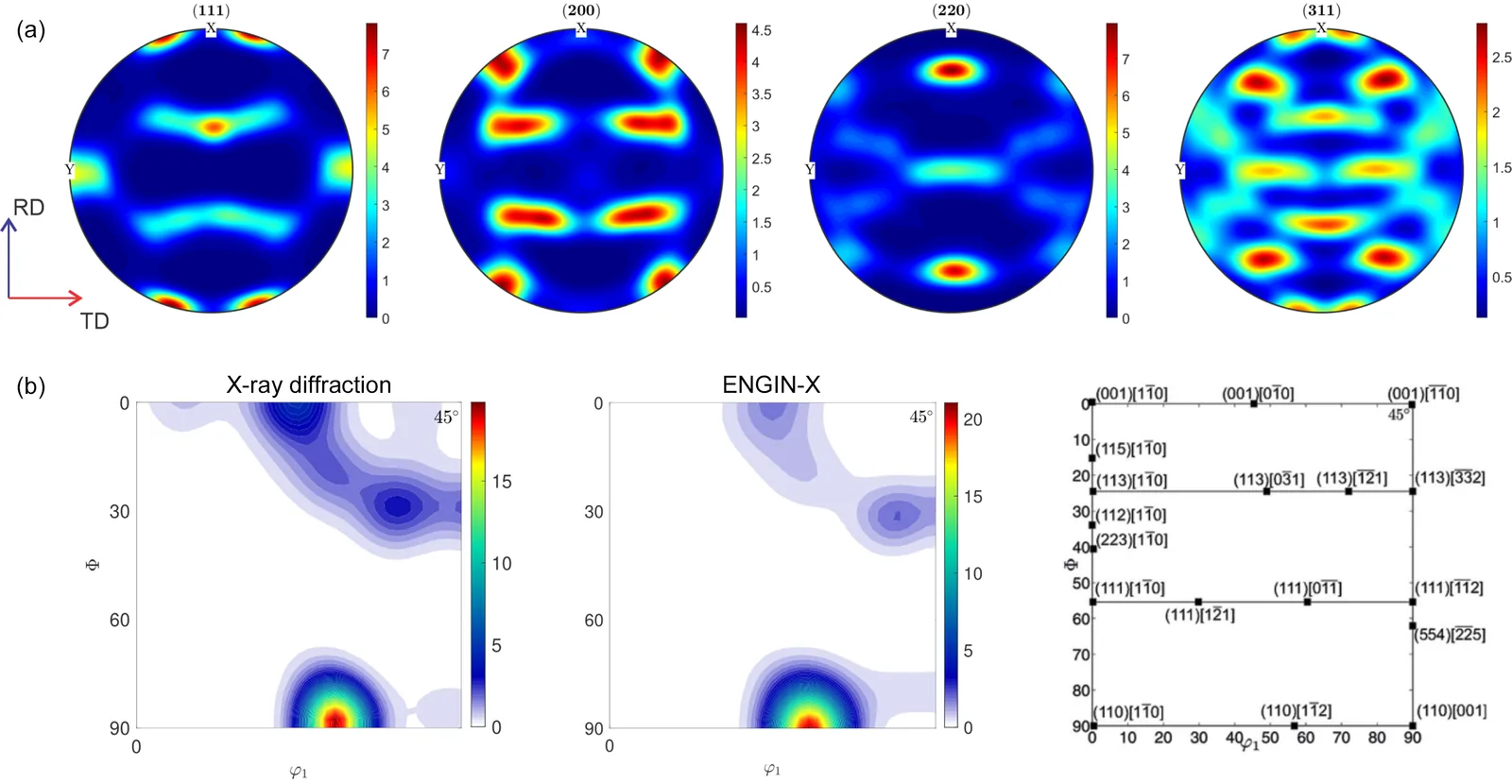
(*a*) Recalculated pole figures from the ODF calculated with *MTEX* from experimental pole figures presented in Fig. 8(*c*). (*b*) Cut of the ODF at φ_2_ = 45°, showing the main texture components of the material obtained by X-ray diffraction experiments and the current work (ENGIN-X).

From the experimental pole figures, the material’s ODF was calculated using the *MTEX Toolbox*, assuming triclinic sample symmetry. Fig. 9 ▸(*a*) shows the recalculated pole figures obtained from the ODF using the 5 min dataset and the 2 × 3 × 5 gridding scheme [Fig. 8 ▸(*c*)], correcting for the minor sample mis-alignment on the goniometer (which can be seen in the offset centres of symmetry on the experimental pole figures). The resulting ODF was calculated with 5° resolution in the orientation space. Comparison with the results obtained with laboratory X-ray diffraction on the same specimen (Malamud *et al.*, 2014 ▸) demonstrated good agreement between the pole figures obtained by the two techniques.

A quantitative comparison between the two techniques can be observed in terms of the volume fraction of the principal texture components of the specimen, as displayed in the cut of the ODF at φ_2_ = 45° [Fig. 9 ▸(*b*)]. Two main components are clearly identified from the plot: a (110)[112] brass component characteristic of rolled aluminium, with a volume fraction of 16% and 18% of the crystallites, and a (001)[010] component with a 1% and 2% volume fraction for the X-ray and ENGIN-X measurements, respectively. The volume fractions were computed by *MTEX* using a 15° radius sphere centred at each orientation. The minor differences observed in the volume fraction are presumably due to some spatial texture inhomogeneity still present in the sample, coupled with the differences in the gauge volume probed by the two techniques.

The close agreement between the ENGIN-X pole figures and the laboratory X-ray results confirms that the shared and newly implemented reduction steps reproduce the established texture for this specimen, rather than introducing systematic bias.

The data analysis pipeline also includes the possibility of using the integrated intensity of the peaks to create experimental pole figures. In the present case, the reconstructed ODF employing the integrated or the fitted peak intensity presented a difference of 3%, calculated by *MTEX* as the normalized *L*2-norm of the difference between the two ODFs, and similar volume fractions of the principal texture components, with discrepancies of less than 10% between the two approaches.

#### 5.3.1. Effect of bank gridding

**Figure 10:**
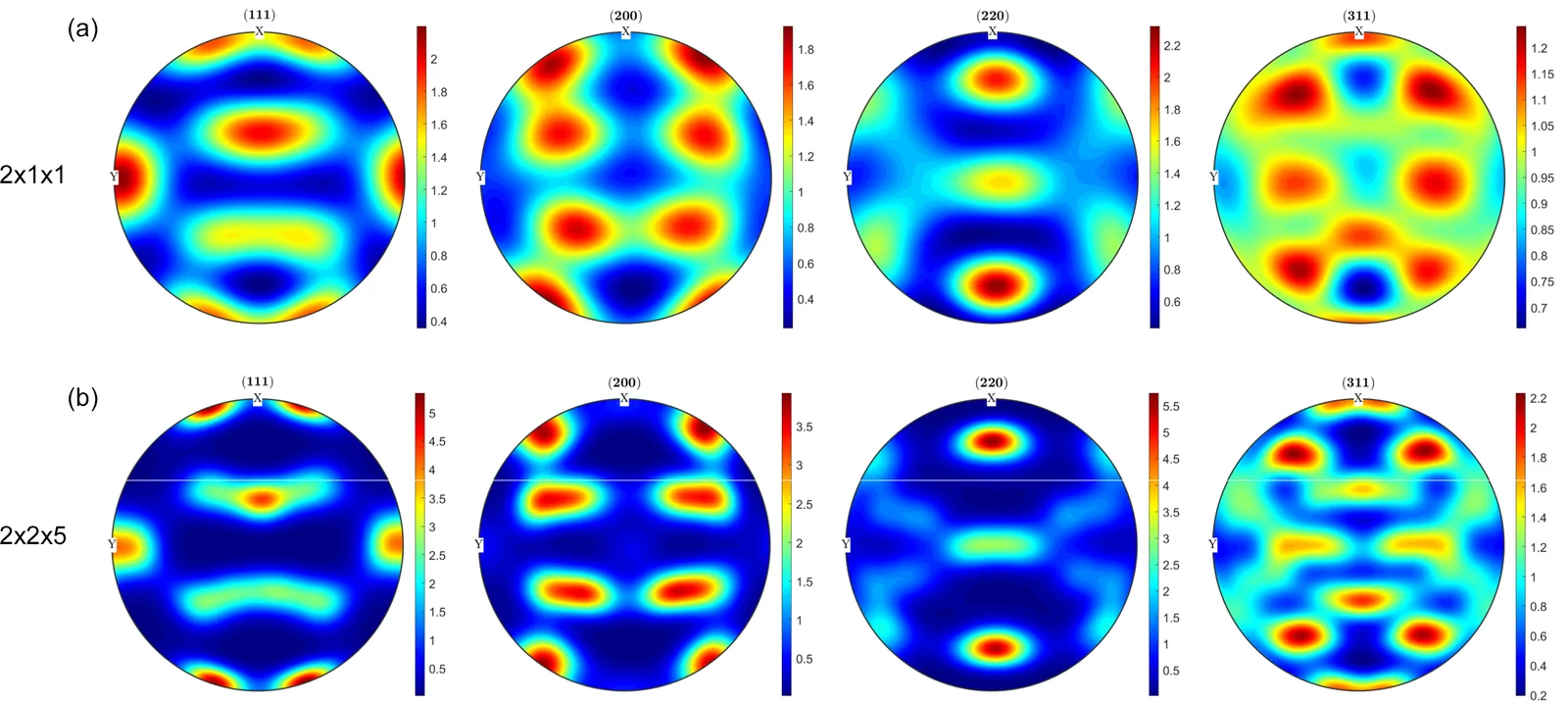
Recalculated pole figures from the ODF calculated with *MTEX* from experimental pole figures using the 5 min dataset employing (*a*) the 2 × 1 × 1 gridding and (*b*) the 2 × 2 × 5 gridding.

The effect of the gridding in the reconstructed pole figure can be seen in Fig. 10 ▸. The coarser 2 × 1 × 1 bank-wide grouping results in a significantly lower angular resolution of the experimental pole figures, which constrains the ODF reconstruction to a kernel half-width of 15° in the *MTEX* estimation. In contrast, the finer 2 × 2 × 5 gridding provides improved pole-figure coverage, enabling a kernel half-width of 6°, resulting in a sharper and better resolved ODF. This is clearly reflected in the recalculated pole figures, where the 2 × 2 × 5 scheme produces more localized and well defined intensity maxima, with intensities reaching up to ∼5.5 mrad in contrast to ∼2.2 mrad for the bank-wide grouping. The broader intensity distributions observed in the 2 × 1 × 1 case are indicative of artificially smoothed texture components, which may lead to an underestimation of the true sharpness of the texture.

These results highlight the importance of an appropriate detector subdivision strategy when performing texture measurements on ENGIN-X, as the angular resolution of the pole-figure coverage directly governs the fidelity of the reconstructed ODF. However, finer detector subdivisions come at the cost of reduced counts per detector group, making the acquisition time a critical parameter to consider, as discussed in the following section.

This resolution/statistics trade off is consistent with that reported for detector-division approaches on other TOF instruments (Kockelmann *et al.*, 2006*a* ▸; Malamud *et al.*, 2014 ▸; Xu *et al.*, 2018 ▸) and at compact sources (Xu *et al.*, 2020 ▸). A key advantage of the present *Mantid* implementation is the flexibility of grouping being applied after collection at the per-pixel level. This allows the balance between statistics and resolution to be explored as a post-acquisition software choice rather than one fixed during the experiment.

#### 5.3.2. Effect of run duration

**Figure 11:**
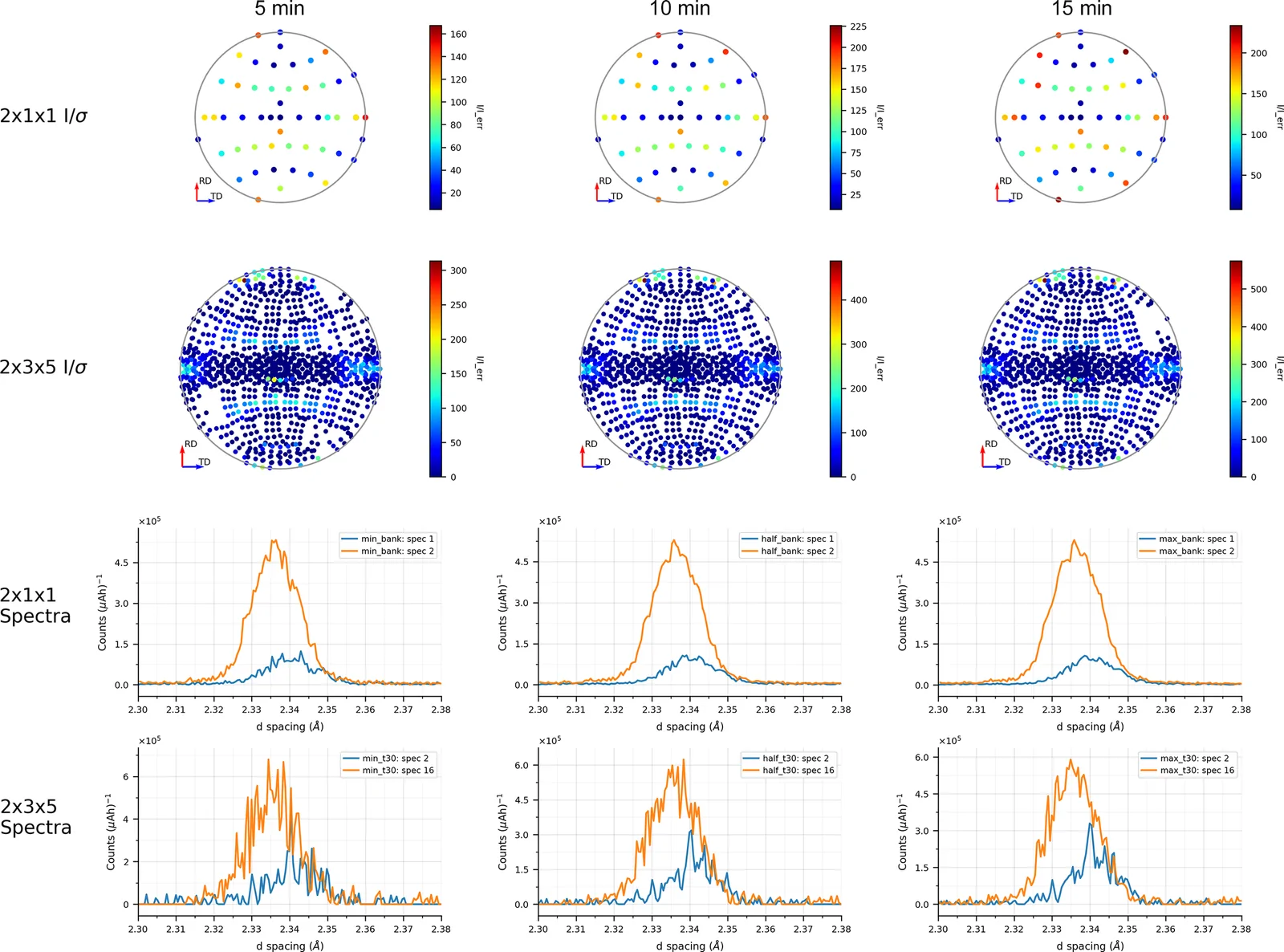
Effect of increasing acquisition time upon the quality of the resulting data. (Top) Fitted intensity divided by intensity error for the 2 × 1 × 1 and the 2 × 3 × 5 virtual detector griddings. (Bottom) Example spectra cropped around the (111) Bragg peak. For the 2 × 1 × 1 spectra both patterns are shown, while for the 2 × 3 × 5 spectra the second and sixteenth patterns are shown, as these detectors are on the fringe of the peak in the ODF, so show appreciable improvement from the increased acquisition time.

The experimental data were collected over a series of three 5 min acquisitions, which allows for effective production of 5 min, 10 min and 15 min representative datasets. Comparing the results after processing these datasets (Fig. 11 ▸) shows that the intensity values obtained are largely independent of the acquisition time (spectra are normalized by current), but subsequent errors on these fitted intensities reduce with time, as expected. This reduced intensity error represents an improved signal-to-noise ratio, which can be seen by comparing the peak and intensity scatter in the figure.

## 6. Summary and outlook

We have developed and implemented a data reduction pipeline for spatially resolved neutron texture analysis within the *Mantid* framework. The workflow processes TOF neutron diffraction data from ENGIN-X, providing an integrated set of tools for experiment planning, absorption correction, detector calibration, peak fitting and experimental pole-figure generation. The pipeline extends the functionality of the previously developed *NyRTeX* software (Malamud *et al.*, 2014 ▸) within *Mantid*, with improved support for large and complex datasets. Interfaces to established texture analysis packages such as *MTEX* are provided for ODF reconstruction and quantitative texture characterization.

The pipeline was validated against a high-strength aluminium alloy sample (AA7150) previously characterized using *NyRTeX*, allowing the *Mantid* results to be checked against the same X-ray reference as was used in the *NyRTeX* validation. The experimental pole figures obtained using the new pipeline show excellent agreement with those of laboratory X-ray diffraction (Malamud *et al.*, 2014 ▸), which confirms the reliability of the workflow. The ODF analysis identified the main texture components of the AA7150 specimen, with volume fractions in close agreement with those reported previously. The effect of detector bank gridding on the reconstructed ODF was investigated, demonstrating that finer virtual detector subdivisions provide a substantially improved pole-figure angular resolution, enabling the use of sharper ODF kernels in the *MTEX* reconstruction (half-widths of 6° versus 15° for the 2 × 2 × 5 and 2 × 1 × 1 groupings, respectively). The effect of acquisition time was also assessed, confirming that longer counting times improve the signal-to-noise ratio of individual detector spectra without introducing systematic biases in the fitted peak intensities, as the data are normalized by proton current. These two parameters, detector grouping and acquisition time, are therefore complementary and their optimal balance for specific samples should be considered during experiment planning.

On the basis of the current workflow, several directions for further development are foreseen. The current implementation focuses on single-phase materials; extension to multiphase systems, where overlapping diffraction peaks require simultaneous fitting, would significantly broaden the applicability of the workflow. Beyond intensity-based pole figures, the pipeline is suited for extension to generalized pole figures. In particular, the peak centre position, related to the lattice *d* spacing, can be used to construct strain pole figures, providing spatially and crystallographically resolved residual stress information. Similarly, the peak width can be mapped to produce microstrain pole figures, offering insight into the directional dependence of lattice defect distributions and deformation-induced broadening. These generalized pole figures would provide a powerful complement to conventional texture analysis, enabling a more complete characterization of the microstructural state of engineering components from a TOF multidirectional diffraction dataset. Finally, the workflow could be adapted for use on other TOF neutron strain scanners, such as POLDI at SINQ, Switzerland, where similar experimental and data analysis tools are employed.

Further information can be found in the *Mantid* documentation at https://docs.mantidproject.org/nightly/concepts/TextureAnalysis.html and https://docs.mantidproject.org/nightly/techniques/TextureReduction.html.

## Supplementary Material

Additional table and figures. DOI: 10.1107/S1600576726007661/xx5106sup1.pdf

Raw Data and processing scripts: https://doi.org/10.5281/zenodo.19551197

## Data Availability

Data files and scripts can be found at https://doi.org/10.5281/zenodo.19551197.
