# Supplementary material for: Development of a neutron texture analysis reduction pipeline in Mantid
Source: J Appl Crystallogr. 2026 Sep 28;59(Pt 5):1595–613. doi: 10.1107/S1600576726007661 (PMC13636036; doi:10.1107/S1600576726007661)
Supplement: Supplementary file 1 [file j-59-01595-sup1.pdf]

JOURNAL OF APPLIED CRYSTALLOGRAPHY

Volume 59 (2026)

Supporting information for article:

Development of a neutron texture analysis reduction pipeline in *Mantid*

Andy Bridger, Winfried Kockelmann, Joe Kelleher, Manuel Morgano, Richard Waite, Sarah Foxley and Florencia Malamud

S1 Supplementary Information

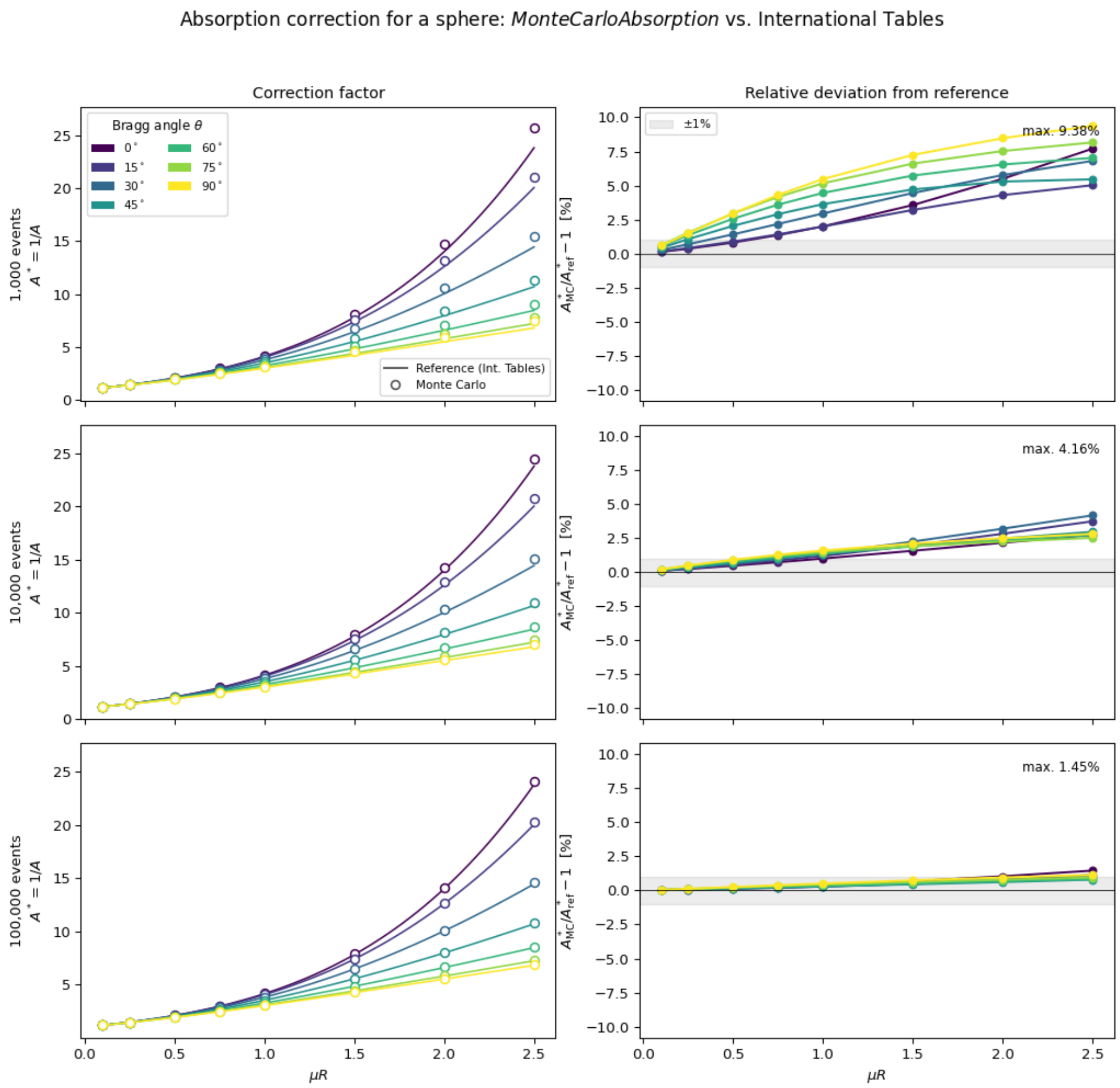

Figure S1: The MonteCarloAbsorption algorithm returns the transmission A (<= 1) here plots A* = (1/A). The path attenuation in MC uses the TOTAL cross section (scatter + absorb). The published tables use the total linear attenuation coefficient μ, so we put all of μ into a *wavelength independent* scattering cross section (such that absorption = 0). With number density N = 1 the identity $\mu = N\sigma$ holds, so for a chosen radius R[cm]: $\sigma_{\text{scat}} = \frac{\mu R}{R}$. Because μ is wavelength independent, A is flat in wavelength. We plot comparison between the reference analytical solution for a sphere (lines, left plots) found in the international tables against the results of the MonteCarloAbsorption algorithm (points, left plots) at a variety of Bragg angles (see colour legend). In the right panel we show the percentage error between the reference and the MonteCarloAbsorption, and here the lines just join those sample points to make the trends easier to see. Vertically we vary the number of path samples used in the simulation and it can be seen that when the sampling of points is sufficiently high the accuracy converges on the analytical result. The main consideration here is that the illuminated volume is sampled sufficiently densely to be representative of the experimental conditions - a criterion which is easily met for texture experiments with a small, defined gauge volume.

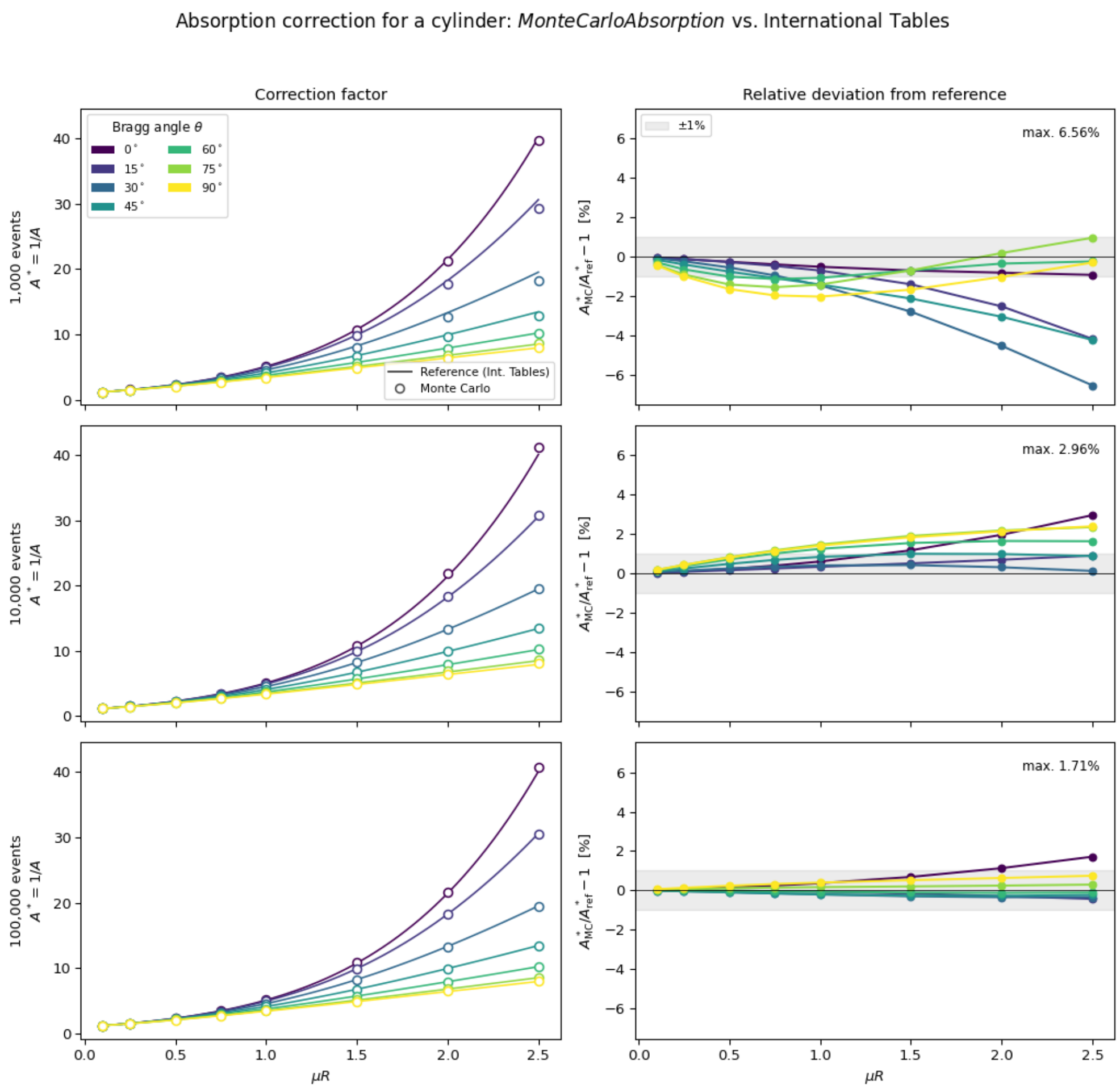

Figure S2: The MonteCarloAbsorption algorithm returns the transmission A (<= 1) here plots A* = (1/A). The path attenuation in MC uses the TOTAL cross section (scatter + absorb). The published tables use the total linear attenuation coefficient μ, so we put all of μ into a *wavelength independent* scattering cross section (such that absorption = 0). With number density N = 1 the identity $\mu = N\sigma$ holds, so for a chosen radius R[cm]: $\sigma_{\text{scat}} = \frac{\mu R}{R}$. Because μ is wavelength independent, A is flat in wavelength. We plot comparison between the reference analytical solution for a cylinder (lines, left plots) found in the international tables against the results of the MonteCarloAbsorption algorithm (points, left plots) at a variety of Bragg angles (see colour legend). In the right panel we show the percentage error between the reference and the MonteCarloAbsorption, and here the lines just join those sample points to make the trends easier to see. Vertically we vary the number of path samples used in the simulation and it can be seen that when the sampling of points is sufficiently high the accuracy converges on the analytical result. The main consideration here is that the illuminated volume is sampled sufficiently densely to be representative of the experimental conditions - a criterion which is easily met for texture experiments with a small, defined gauge volume.

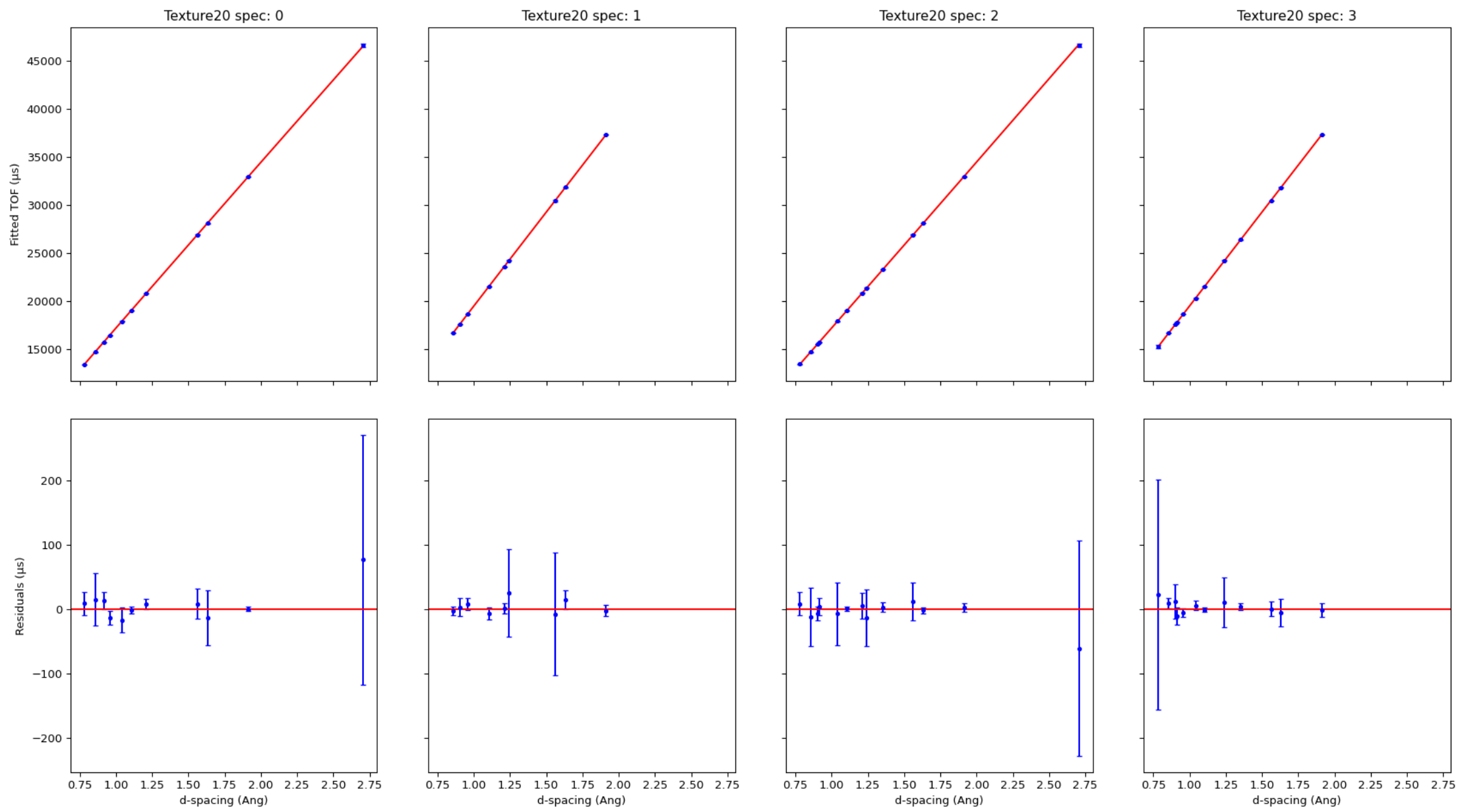

Figure S3: Results of the CeO_2 calibration fits for conversion between TOF and d spacing.

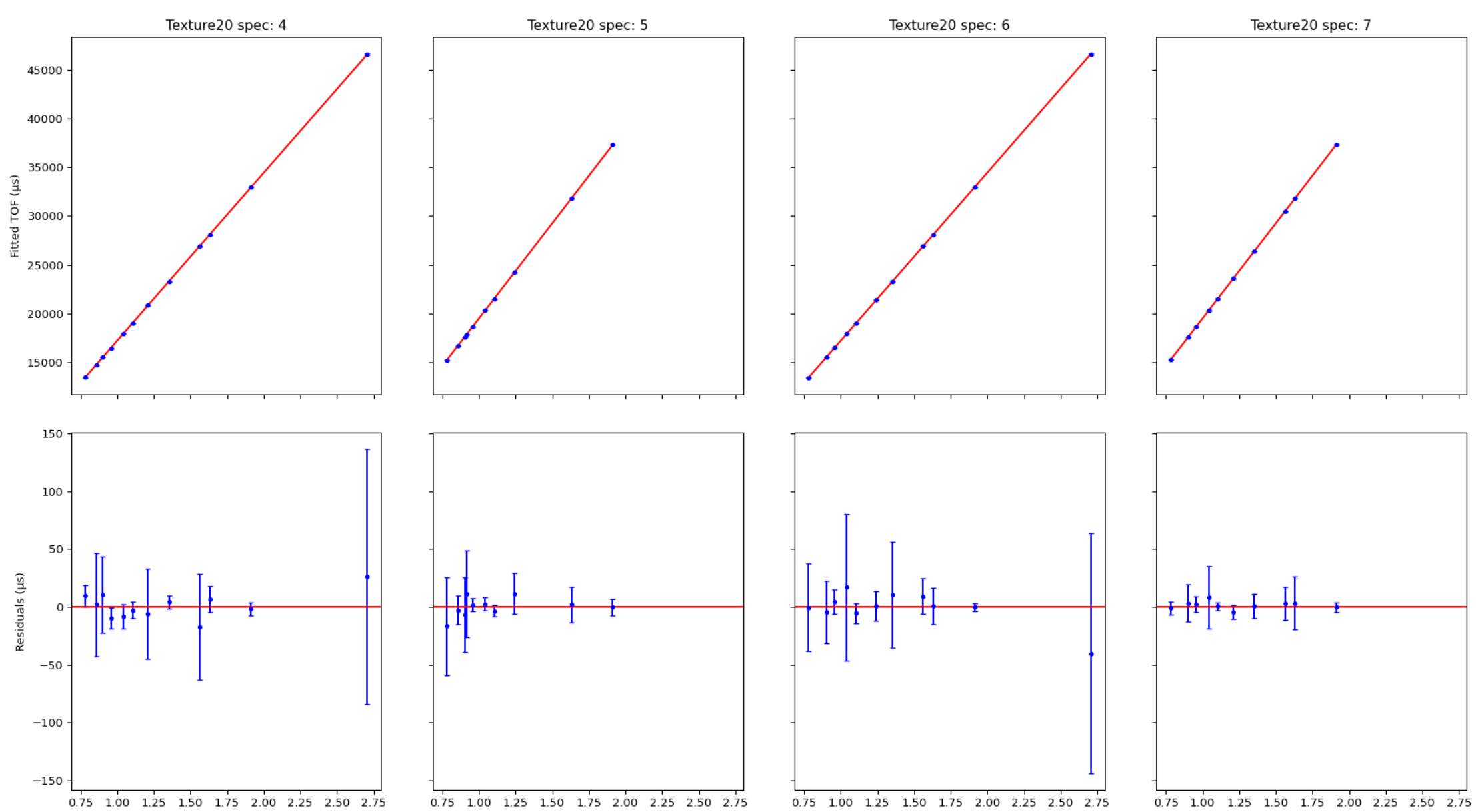

Figure S4: Results of the CeO_2 calibration fits for conversion between TOF and d spacing.

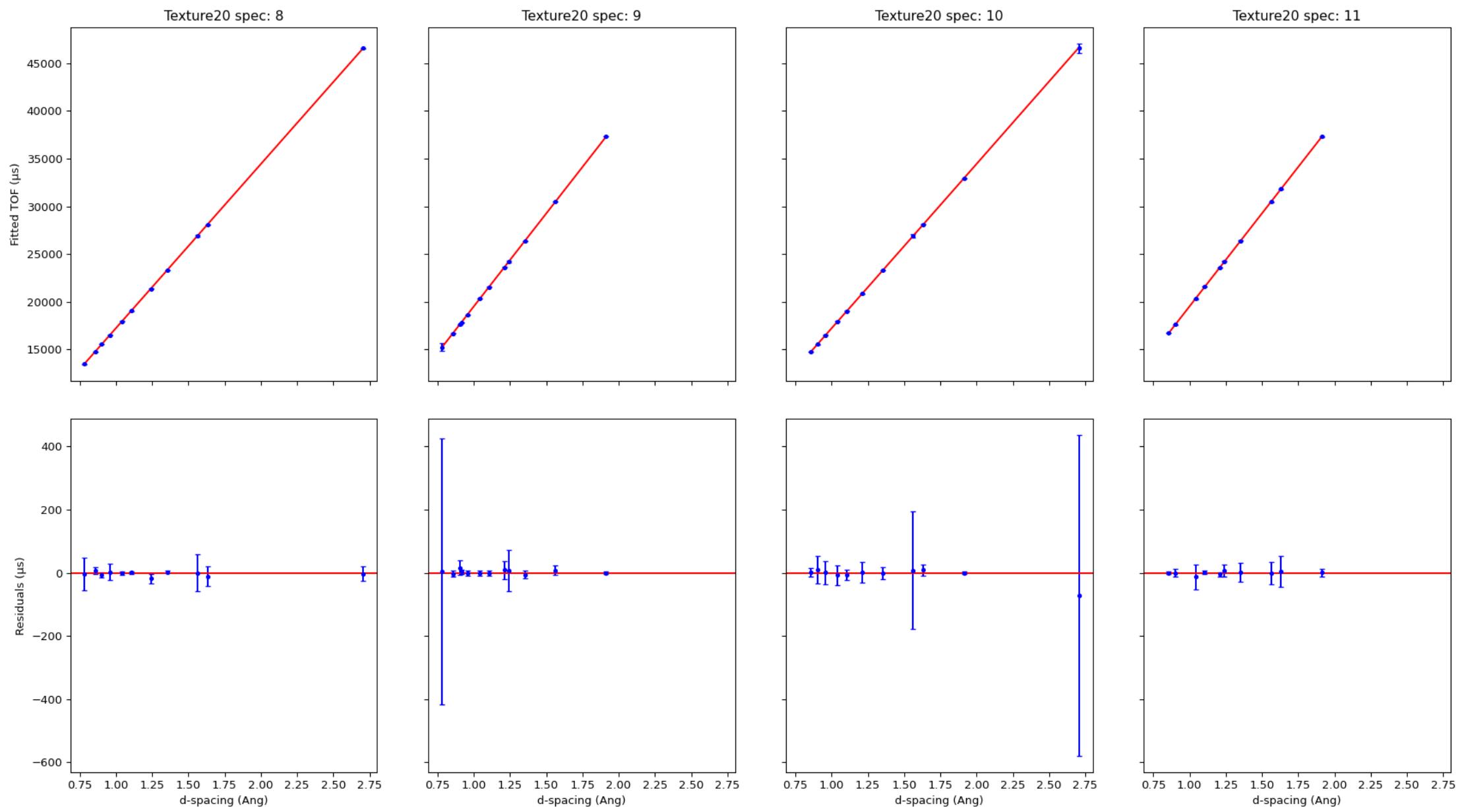

Figure S5: Results of the CeO_2 calibration fits for conversion between TOF and d spacing.

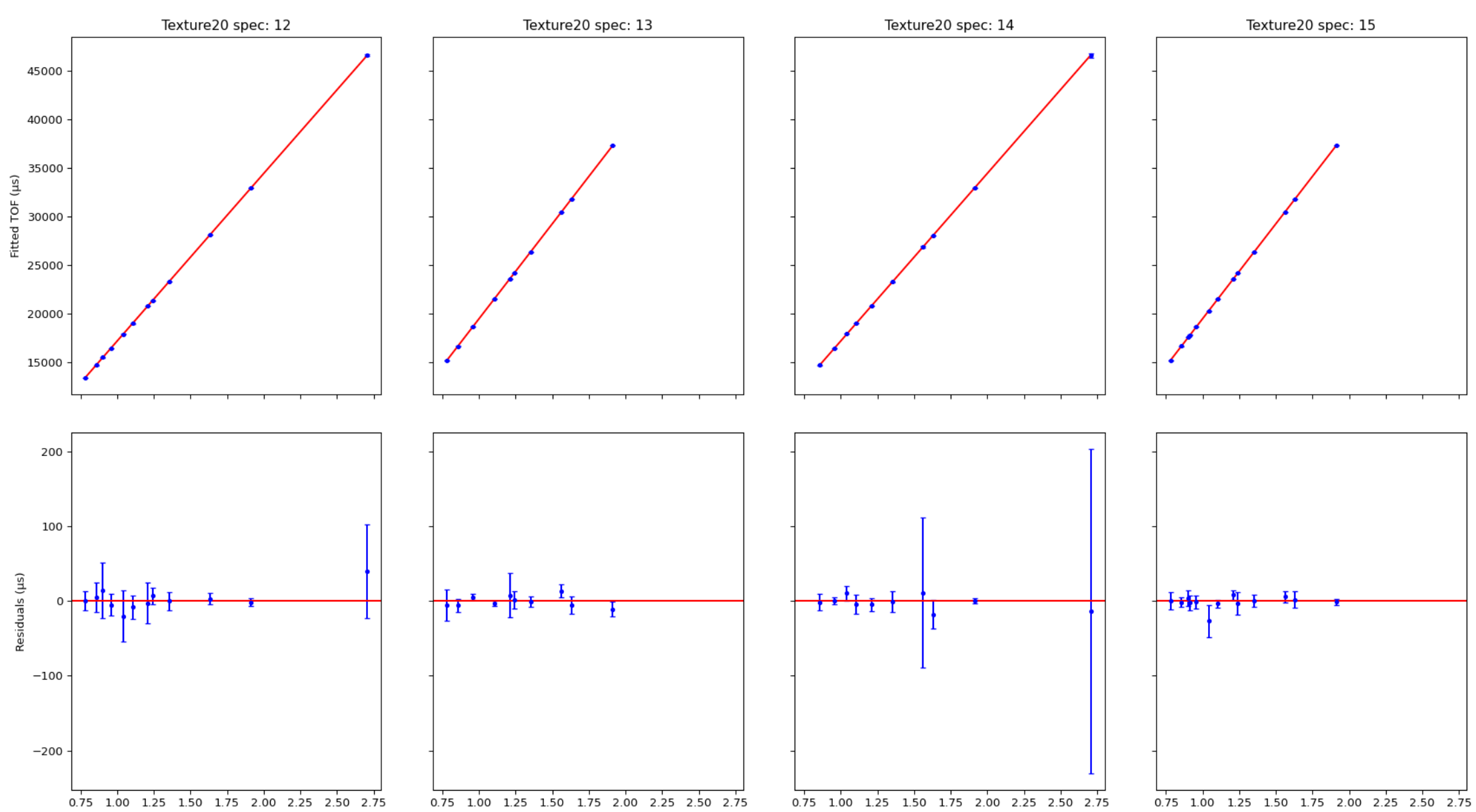

Figure S6: Results of the CeO_2 calibration fits for conversion between TOF and d spacing.

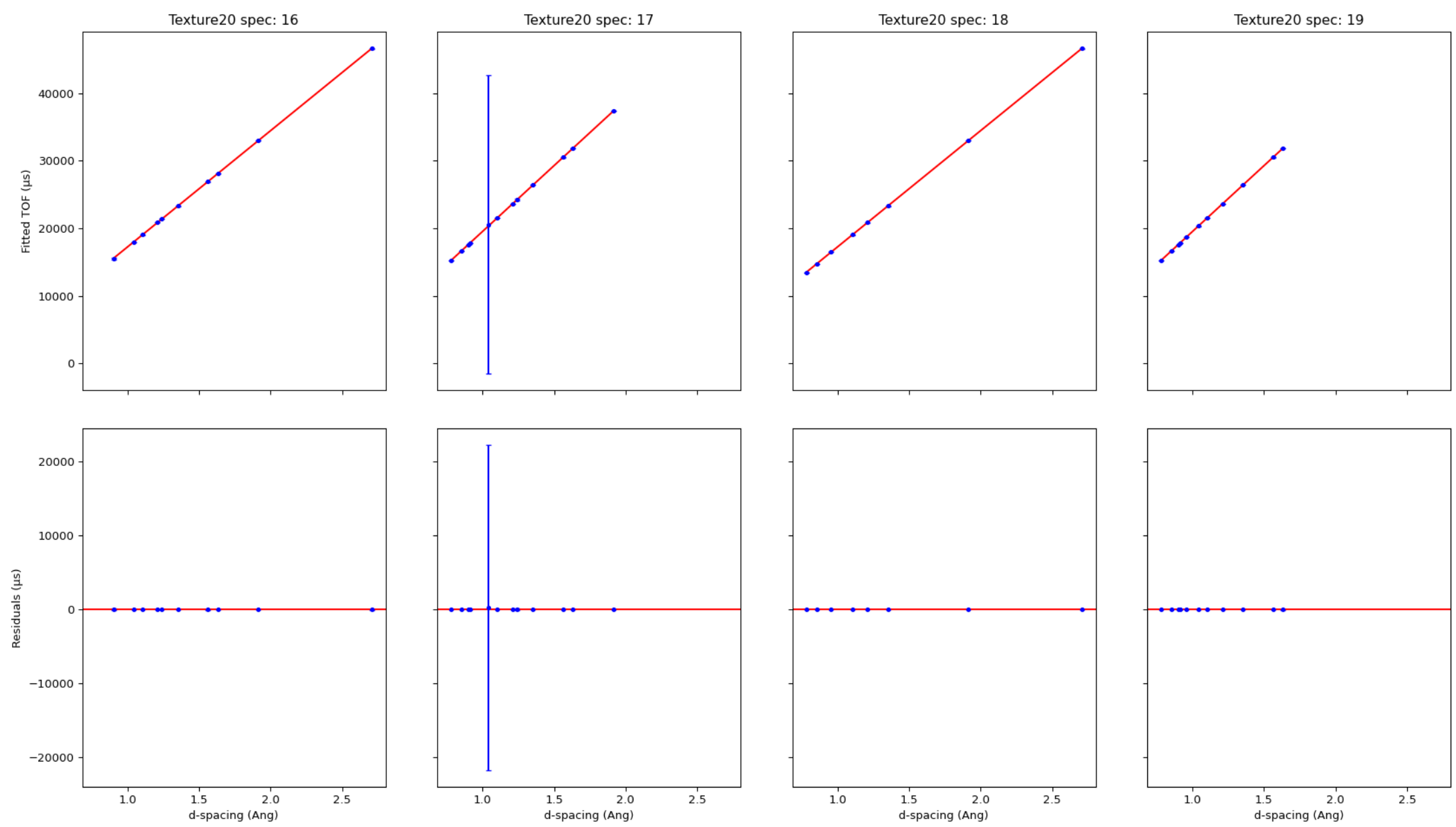

Figure S7: Results of the CeO_2 calibration fits for conversion between TOF and d spacing.

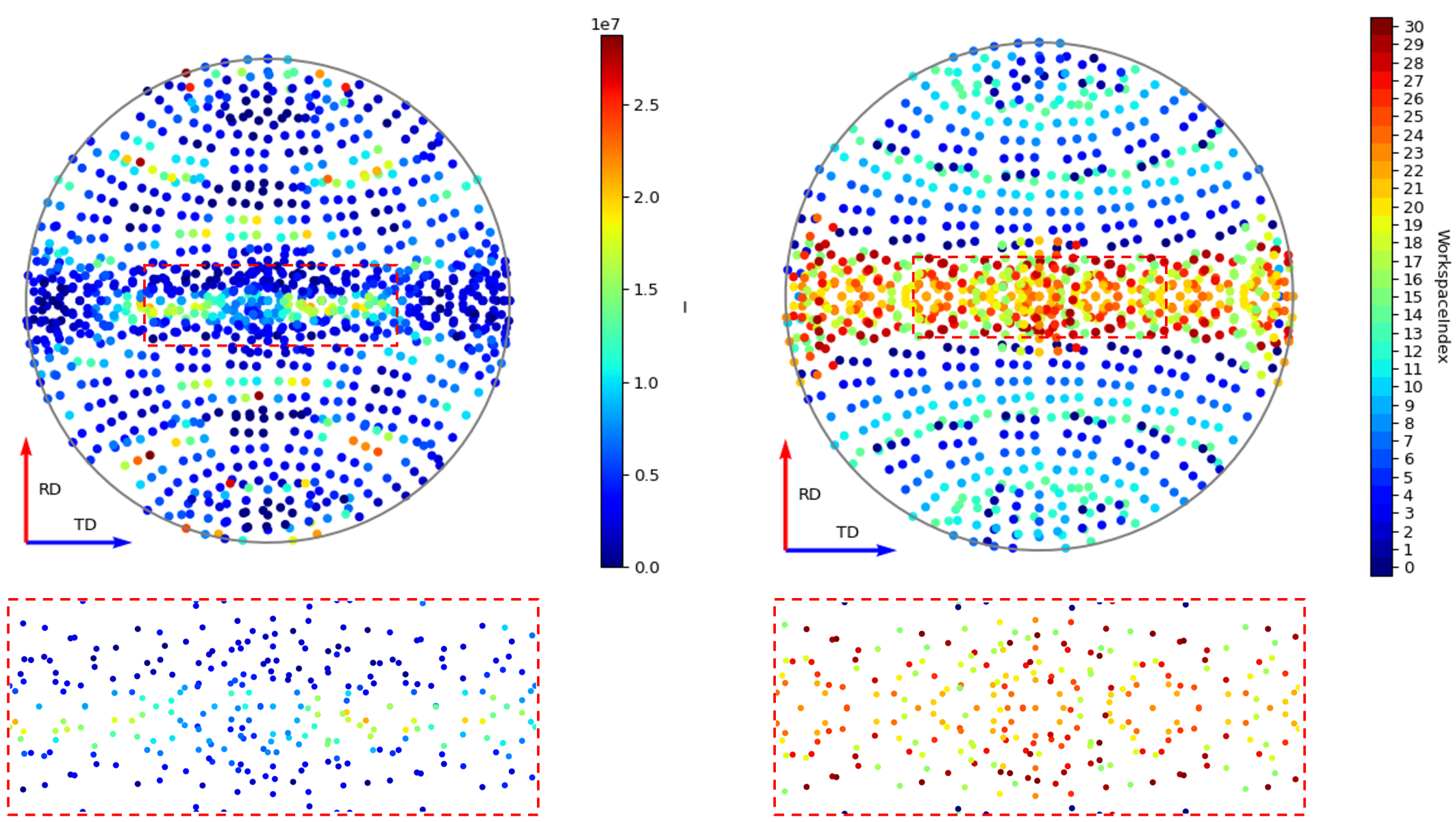

Figure S8: Figure shows that regions which give consistent results for peak texture are produced from data points sampled in different pixel modules and thus intensity extracted from peaks recorded at different TOFs.

Table S1: Rotation angles used for the texture measurements. Note: these are intrinsic angles, so the axes rotate with the previous rotations (Rot2 is about axis Z', the Z axis after a rotation, Rot1, about Y. Rot3 is about axis Y", the Y axis after rotation Rot2 about Z').

Rot1 (Y)	Rot2 (Z')	Rot3 (Y")
45	0	0
45	30	0
45	30	20
45	30	40
45	30	60
45	30	80
45	30	100
45	30	120
45	30	140
45	30	160
45	30	180
45	30	200
45	30	220
45	30	240
45	30	260
45	30	280
45	30	300
45	30	320
45	30	340
45	55	10
45	55	40
45	55	70
45	55	100
45	55	130
45	55	160
45	55	190
45	55	220
45	55	250
45	55	280
45	55	310
45	55	340
45	90	0
45	90	90
30	90	0
30	90	90
30	90	180
30	90	270

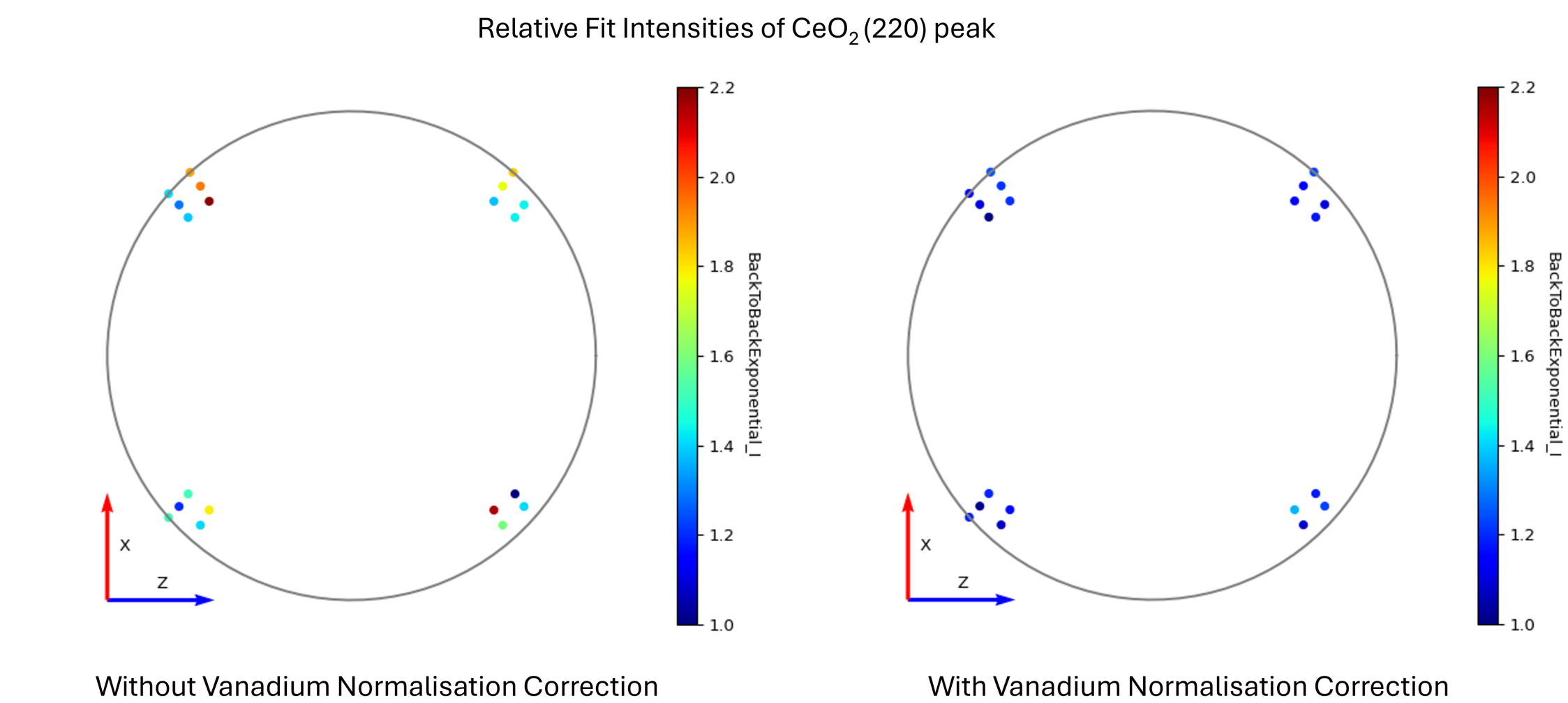

Figure S9: Figure shows the effect of the vanadium normalisation on the texture of the reference CeO_2 powder. Applying the normalisation reduces the strength of the apparent texture considerably, however, not perfectly, as not absorption correction has been applied nor has a quantification been made of how explicitly untextured the CeO_2 sample is (powder intended to be homogeneous enough to give some intensity in all pixels not uniformly untextured).
